# To Reconstruct or Discard: A Comparison of Additive and Subtractive Charge Sharing Correction Algorithms at High and Low X-ray Fluxes

**DOI:** 10.3390/s24154946

**Published:** 2024-07-30

**Authors:** Oliver L. P. Pickford Scienti, Dimitra G. Darambara

**Affiliations:** Joint Department of Physics, Institute of Cancer Research and Royal Marsden NHS Foundation Trust, London SM2 5NG, UK; dimitra.darambara@icr.ac.uk

**Keywords:** energy resolving, photon counting, spectral imaging, CdTe, charge sharing correction, subtractive CSCA, additive CSCA, x-CSI, optimisation

## Abstract

Effective X-ray photon-counting spectral imaging (x-CSI) detector design involves the optimisation of a wide range of parameters both regarding the sensor (e.g., material, thickness and pixel pitch) and electronics (e.g., signal-processing chain and count-triggering scheme). Our previous publications have looked at the role of pixel pitch, sensor thickness and a range of additive charge sharing correction algorithms (CSCAs), and in this work, we compare additive and subtractive CSCAs to identify the advantages and disadvantages. These CSCAs differ in their approach to dealing with charge sharing: additive approaches attempt to reconstruct the original event, whilst subtractive approaches discard the shared events. Each approach was simulated on data from a wide range of x-CSI detector designs (pixel pitches 100–600 µm, sensor thickness 1.5 mm) and X-ray fluxes (10^6^–10^9^ photons mm^−2^ s^−1^), and their performance was characterised in terms of absolute detection efficiency (ADE), absolute photopeak efficiency (APE), relative coincidence counts (RCC) and binned spectral efficiency (BSE). Differences between the two approaches were explained mechanistically in terms of the CSCA’s effect on both charge sharing and pule pileup. At low X-ray fluxes, the two approaches perform similarly, but at higher fluxes, they differ in complex ways. Generally, additive CSCAs perform better on absolute metrics (ADE and APE), and subtractive CSCAs perform better on relative metrics (RCC and BSE). Which approach to use will, thus, depend on the expected operating flux and whether dose efficiency or spectral efficiency is more important for the application in mind.

## 1. Introduction

Photon-counting (PC) X-ray techniques are widely recognised as the future of X-ray imaging due to their ability to leverage spectral information, in addition to the attenuation differences measured by conventional X-ray scanners, in a more dose-efficient way. This additional information can be used in a wide range of industrial and clinical applications [1], including contrast agent quantification [2], disease assessment [3], implant assessment [4] and tissue differentiation [5,6]. Focussing specifically on medical applications, PC techniques have proven suitable for identifying various metallic nanoprobes [7,8], with the promise of transforming X-ray imaging into a molecular imaging modality which could have implications for fields such as oncology, allowing tumour cells to be identified based on the cell surface receptors that they express [9,10].

PC systems work by counting the number of X-ray photons arriving at the detector rather than the total energy they deposit. To do this, PC uses fast electronics (timing resolution on the order of nanoseconds) to count whenever the charge in the detector rises above some preset threshold. By setting this threshold above the noise floor of the system, the PC is able to almost completely suppress electronic noise [11], and the counting approach improves soft tissue contrast by weighting photons evenly rather than proportionally to their energy [12]. Combined, these features allow for much smaller pixels to be employed in PC systems, allowing for significantly improved spatial resolutions and lower doses [13,14]. 

An advance on simple PC techniques involves using multiple threshold–counter pairs to group the detected X-rays based on their energy, e.g., an X-ray may be recorded as having energy above threshold 1 but below threshold 2, etc. This approach is generally referred to as X-ray photon-counting spectral imaging (x-CSI) or spectral photon-counting computed tomography (SPCCT) when performed in 3D. x-CSI allows for a far more flexible weighting of the X-rays based on their energy and provides spectral information, improving material decomposition approaches and allowing for simultaneous quantification of multiple contrast agents [2,5,6,7,8,9,10,11,12,13,14,15,16,17].

x-CSI still has limitations, primarily associated with maintaining spectral fidelity at sufficiently high count rates for clinical applications without compromising spatial resolution. The trade-off between spectral fidelity and spatial resolution is due to a range of processes [18] (Compton scattering, X-ray fluorescence and charge cloud expansion), which collectively mean that X-rays can deposit their energy over an area of several hundred µm in the sensor. These processes, referred to as charge sharing effects (CSEs), mean that as pixel sizes decrease, the proportion of X-rays, which will deposit their energy across multiple pixels, increases. Where this happens, the recorded energy spectrum could be distorted in several ways [19,20,21]: recording multiple counts when only a single photon arrived; recording these counts at an energy lower than the actual photon that arrived; and incorrectly recording the location of the primary photon interaction with the sensor.

We have previously published on how CSEs affect the performance of x-CSI systems, first considering which sensor geometries are most susceptible to them [20] and then comparing a range of proposed charge sharing correction algorithms (CSCAs) for their ability to address CSEs [22]. This paper is the third paper in that series in which we compare the behaviour of two major classes of CSCA (additive and subtractive) as a function of X-ray flux. This is performed to determine which type of CSCA would be expected to perform better for a range of medical applications.

It is expected that this work will prove a useful reference point for guiding interested researchers and x-CSI manufacturers towards selecting the most appropriate CSCA for their application, as well as motivating research on better CSCAs in the future.

## 2. Materials and Methods

The work presented here was simulated using CoGI (**Co**msol **G**ate **I**nterlocutor), our in-house x-CSI simulation framework [23]. As this paper is the third part in a series, we refer the interested reader to our previous publications for more detailed information regarding the working of CoGI [20,22]. In this paper, however, we will provide a brief overview of CoGI and details of how it was modified for the work described here.

**Figure 1:**
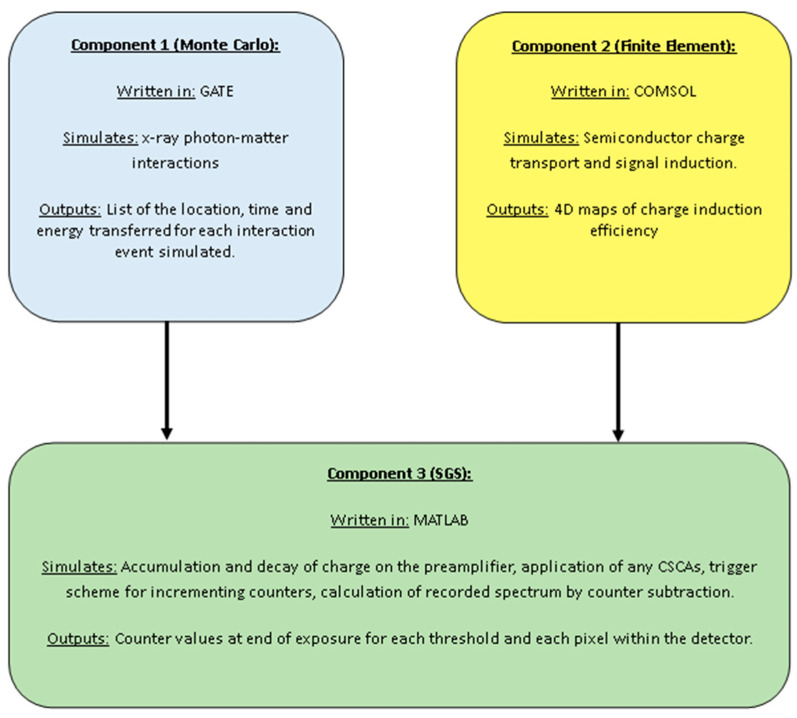
Diagram showing the components of CoGI, simulation tools they employ and data flow between them.

CoGI is comprised of three components, as shown in Figure 1. The first component is a Monte Carlo code, executed in GATE version 7 [24], which handles the processes of X-ray interaction with matter, energy deposition and inter-pixel charge transport caused by X-ray fluorescence and Compton scattering.

The second component is a finite element model, implemented in COMSOL [25], which solves Prettyman’s adjoint continuity equations [26] to calculate the charge induced in the collecting anodes as a result of intra-pixel charge transport. Specifically, the processes of diffusion, drift and trapping are modelled for each possible initial charge cloud location. As a result of this implementation, the charge which is transported across pixel boundaries by these processes is assumed to be lost rather than shared, which is a limitation of the current model. On this limitation, it is important to note that CoGI was successfully validated using a physical x-CSI detector with 100 µm pitch with very good agreement [23]. Whilst smaller pixel pitches have been proposed for x-CSI applications [27], charge sharing effects begin to dominate at pitches below ~100 µm, so we limited our simulations to pixels greater than this size. 

The final component is a series of custom scripts written in Matlab R2022a [28], which handles data curation and processing. First, this component combines the output files from the Monte Carlo and finite element model programs to produce a list of induced charges for each pixel as a function of time: the raw signals. Any selected CSCAs are then applied to these raw signals, and the specified threshold–counter pairs are simulated. Finally, an image and summed energy spectra are produced, allowing for a range of system metrics to be calculated (see Section 2.6 below).

The parameters implemented in each component are detailed in the following sections.

### 2.1. Component 1 Parameters

The sensor comprised a single CdTe crystal of cross-sectional area 21 mm × 21 mm and a thickness of 1.5 mm. This thickness was chosen based on results from our earlier work, showing this thickness to provide a good trade-off between detection efficiency and spectral fidelity [20] and is comparable to the 1.6 mm CdTe sensors used by Siemens [27]. A flat-field irradiation of this sensor was simulated, using a monoenergetic (80 keV), square X-ray source of size 24 mm × 24 mm, with their centres coaligned. The X-ray energy was selected as it is high enough to provide good separation of spectral features in the detected spectra (main photopeak, escape peak and Cd X-ray fluorescence peaks) whilst still being within the range of energies used in medical X-ray imaging.

In total, 4 versions of this simulation were performed, corresponding to X-ray fluxes of 10^6^, 10^7^, 10^8^ and 10^9^ photons mm^−2^ s^−1^. An irradiation time of 10 ms was simulated for the 10^6^ photons mm^−2^ s^−1^ case, and the irradiation time was varied between simulations to keep the total number of photons simulated constant, as shown in Table 1. These times were all several orders of magnitude greater than the shaping time of the systems, allowing sufficient photons for pulse pileup to be modelled. The same random seed was used for each simulation, ensuring the various CSCAs could be compared with the same incident photon distribution.

### 2.2. Component 2 Parameters

The properties of the CdTe material simulated in the finite element models are listed in Table 2. In addition to these parameters, values needed for the simulation were pixel pitch, anode size and bias voltage. Pixel pitch was varied between 100 µm and 600 µm in 50 µm steps, covering the range of pixel pitches currently under research in the x-CSI community. CoGI was originally validated using a physical x-CSI detector provided to us on loan by Varex Imaging [29], and the voltage bias was calculated for the simulated sensor thickness so that the transit time across the pixel would be the same as in this prototype, allowing for a consistent shaping time to also be used. Similarly, the anode size was calculated for each pixel pitch so that the inter-anode spacing would remain consistent with the detector used for our validations. Due to a non-disclosure agreement with Varex Imaging, we are unable to give the exact values used for anode spacing or bias voltage in these simulations. 

### 2.3. Component 3 Parameters

Component 3 takes in the 4 output files from Component 1 and uses them to create 44 pixelated data sets (each X-ray flux with the sensor pixelated at each of the required pitches). The locations of each interaction are thus converted from a global spatial coordinate system to an intra-pixel coordinate system whilst retaining their energy deposition and timing information. Maps from Component 2 relating the location of interaction to charge induction efficiency are then used to determine the height and time of signal pulses that each pixel in the sensor would experience. Component 3 then loops over this data set, implementing a different one of the CSCAs investigated on each pass and storing that output separately. The details of the CSCAs modelled can be found in the next sub-section. The list of CSCA corrected events is then compared with the thresholds calculated, as described in Section 2.5, to determine which counters to increment. The counts on these counters at the end of the irradiation are then used to calculate the energy spectrum for each pixel. The spectra for all pixels were aggregated to allow for the performance metrics listed in sub-Section 2.6 to be calculated for each system as a whole.

### 2.4. Electronics and Data Processing Simulations 

CSCAs operate by searching for events which are separated by a small distance in both space and time and tagging these as potential charge sharing groups (PCSGs). The idea behind this is that it is more probable that such closely spaced events are the result of a single X-ray triggering adjacent pixels than of two unrelated X-rays arriving in adjacent pixels in quick succession. We classify CSCAs into one of two groups, depending on how they deal with PCSGs. These two groups, referred to as their correction method (CM), are ‘additive’ and ‘subtractive’.

Additive CSCAs attempt to reconstruct the energy of the initial X-ray based on the signals recorded in the various pixels. In the CSCAs modelled here, this means summing the charge on each pixel in the PCSG before presenting it to the threshold–counter pairs in the pixel with the highest individual charge amongst the PCSG.

In contrast, subtractive CSCAs reduce the impact of charge sharing by rejecting events associated with a PCSG. This approach is expected to prevent the spectral and spatial distortions that can result from incorrect reconstruction in the additive approaches but comes at the cost of reduced counting efficiency.

The additive CSCAs considered in this work are described in more detail in our previous work [22]. Briefly, the CSCAs are all classified by two factors: Neighbourhood Size and Neighbourhood Locality.

Neighbourhood Size

**Figure 2:**
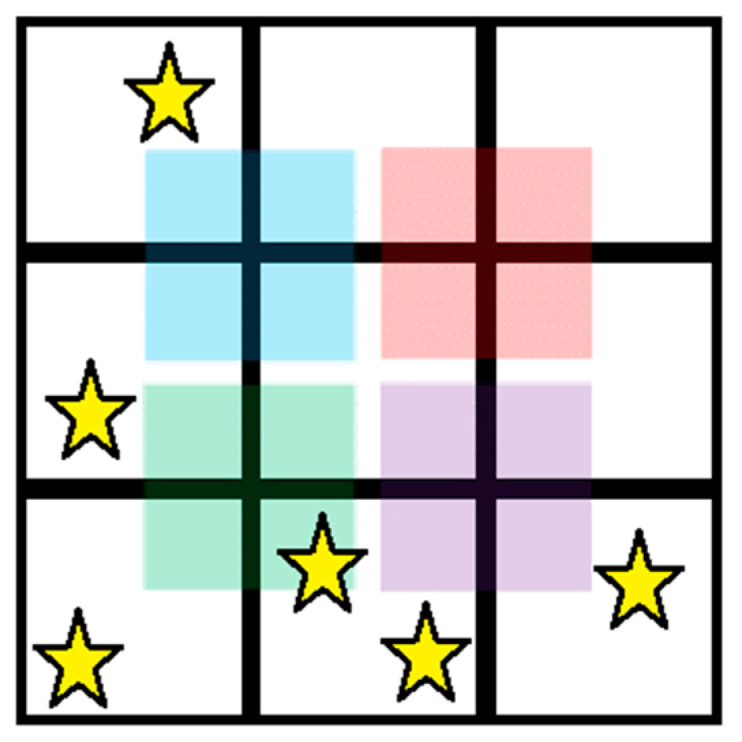
Hybrid CSCAs work by first constructing a 3 × 3 pixel search area, here shown containing multiple detected charges (stars). Next, four 2 × 2 sub-regions are defined, indicated here by coloured squares, which overlap the pixels within their domain. Additive CSCAs compare the highest value of all sub-regions to counter thresholds to determine whether or not to count, whilst subtractive CSCAs compare the lowest value of the sub-regions. In this example, therefore, the additive CSCA would output the charge from pixels covered by the green square (4 stars of charge), whilst the subtractive algorithm would output the charge from the pixels covered by the red square (0 stars of charge, and so no counters are incremented).

Neighbourhood Size (NS) refers to the size of the search area (neighbourhood) used to find PCSGs. NS values used were as follows:

- 2 × 2: to be part of the same PCSG, events must lie within a square neighbourhood of side length 2 pixels;
- 3 × 3: to be part of the same PCSG, events must lie within a square neighbourhood of side length 3 pixels;
- Hybrid: a two-step process, where, first, a 3 × 3 pixel group is used to identify PCSGs, and then, all possible subgroups of 2 × 2 pixels within the 3 × 3 area are compared, with the largest signal set as the single output from the 3 × 3 group (see Figure 2). For more details, see [22].

Neighbourhood Locality

**Figure 3:**
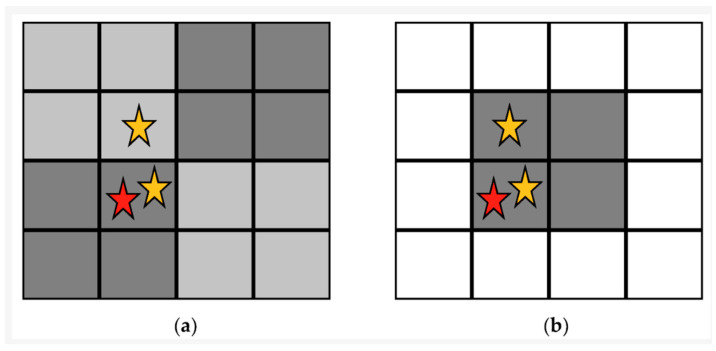
Representation of a 4 × 4 grid of pixels containing 3 detected events (stars), the first of which is marked in red. The events can then be assessed in one of two ways: (**a**) Static algorithms predefine which pixels will be grouped into search areas (grey shading) at the beginning of the image acquisition, and these do not change. (**b**) Dynamic algorithms group pixels into search regions as and when events are detected, according to some geometric rule. In the example shown, the rule is that a detected event (red star) begins a search for other events in the squares to the neighbouring pixels in the east, north and northeast directions. Figure reproduced from [22].

Neighbourhood Locality (NL) refers to the method by which neighbourhoods are defined. Static CSCAs assign each pixel to a fixed neighbourhood at the start of the simulation (Figure 3a). Dynamic CSCAs define neighbourhoods temporarily after each event is detected, based on some fixed geometric rule (Figure 3b).

We then produced subtractive versions of each of these CSCAs. Subtractive CSCAs prevent signals from incrementing counters if multiple signals are detected within the search area at the same time. Again, the Hybrid CSCA is treated slightly differently. The subtractive Hybrid CSCA works in a very similar way to the additive Hybrid CSCA shown in Figure 2, except that its output is equal to the smallest signal found in the 2 × 2 sub-groups rather than the largest.

Combining the three parameters in the form of NS-NL-CM gives each CSCA a unique label; for example, 2 × 2 St+ would be a CSCA with a 2 × 2 neighbourhood size, assigned statically (St) at the simulation start-up, which corrected for PCSGs via an additive (+) mechanism.

### 2.5. Selecting Energy Bin Thresholds

As mentioned previously, the monoenergetic irradiation is expected to produce 3 distinct spectral features: a full-energy photopeak, an escape peak and X-ray fluorescence. Additionally, pulse pileup could be expected to be the sole source of any counts above the photopeak. In order to isolate these spectral features whilst sticking to a realistic number of energy thresholds based on existing technology, thresholds were set, as shown in Table 3.

### 2.6. Performance Metrics Assessed

Four performance metrics were calculated for each system using the aggregate counts from pixels across the detector. These metrics were the same as used in our previous investigations in this series and are reproduced below.

Absolute detection efficiency (ADE) is a measure of how accurately a detector measures the incoming X-ray flux. It is calculated according to Equation (1).

(1) $$ADE=\sum_{E=1}^{4}\frac{B_{E}}{I}$$

where *B_E_* is the count in the *E*th energy bin, and *I* is the number of X-rays incident on the detector.

Absolute photopeak efficiency (APE) is a measure of how often an incident photon is detected and assigned to the correct energy bin. It is calculated according to Equation (2).

(2) $$APE=\frac{B_{3}}{I}$$

where *B_3_* is the number of counts in energy bin 3, and *I* is, again, the number of X-rays incident upon the detector. 

Relative coincidence counts (RCC) are a measure of the pulse pileup experienced by the system. RCC is calculated according to Equation (3).

(3) $$RCC=\frac{B_{4}}{\sum_{E=1}^{4}B_{E}}$$

where *B*_4_ is the number of counts in the 4th energy bin.

Binned spectral efficiency (BSE) is a measure of how often a detected photon is assigned to the energy bin corresponding to its true energy. It is defined according to Equation (4).

(4) $$BSE=\frac{B_{3}}{\sum_{E=1}^{4}B_{E}}$$

where the meanings of the symbols are as defined in Equations (1)–(3).

### 2.7. Analytical Model of Idealised Absolute Detection Efficiency

Optimal performance as measured by APE, RCC or BSE metrics is associated with either maximising or minimising the metric score (0% RCC or 100% APE/BSE). In contrast, the ADE score of an ideal system is somewhere between these extremes, as system imperfections can push ADE both artificially low (due to pulse pileup) or artificially high (due to charge sharing effects). Consequently, when assessing detector performance using ADE, we need to compare to an idealised point of reference. We modelled the behaviour of an idealised detector using the analytical system shown in Equation (5).

(5) $$ADE=P_{I}e^{-F_{det}At_{w}P_{I}}$$

where *F_det_* is the X-ray flux on the detector; *A* is the area of a pixel; *t_w_* is the time required to resolve two events as distinct, and *P_I_* is the probability of an X-ray entering the detector interacting with it photoelectrically. *P_I_* can be calculated from Equation (6).

(6) $$P_{I}=1-e^{-M\rho T}$$

where *M* and ρ are the mass attenuation coefficient and density of CdTe respectively, and *T* is the sensor thickness. 

A full derivation of this analytical model can be found in our previous publication [22]. Briefly, this analytical model assumes that X-rays on a given pixel are separated in time according to a Poisson distribution; all interactions are fully photoelectric (no Compton scattering), and, once deposited, charge is not shared between pixels (no X-ray fluorescence or cloud expansion across pixel borders), and all charge deposited into a pixel is read out (no charge trapping or ballistic deficit). It then calculates the rate at which events arrive with a time separation greater than the shaping time of the detector (allowing them to be resolved in this setup). No CSCAs are applied.

The analytical model, thus, represents a paralysable detector, whilst the simulations performed in CoGI modelled a detector with a force resetting of the electronics between events. Though the ideal behaviours of paralysable and non-paralysable systems will differ at extremely high fluxes, the difference between them should be minor at the majority of fluxes considered in this work. For this reason, the ADE value calculated by this method will be used as a benchmark for an “ideal” detector in the analysis.

### 2.8. Division of Results for Analysis

**Figure 4:**
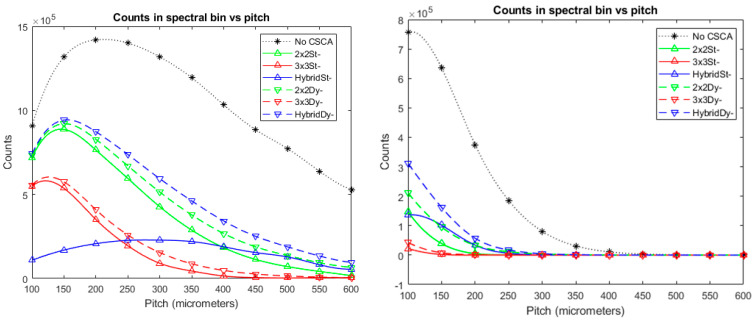
At sufficiently high per-pixel X-ray fluxes, the time between events becomes too small for the sensor to discharge fully, resulting in detector paralysis and, thus, no more counts are recorded. At 10^9^ photons mm^−2^ s^−1^ (**Right**), this happens for almost all simulated detector designs, making meaningful comparisons difficult. In contrast, at 10^8^ photons mm^−2^ s^−1^ (**Left**), there are still sufficient counts recorded at all pixel pitches for the CSCA performances to be differentiated. The figures shown are for the subtractive CSCAs; however, the same situation can be seen in the additive CSCA data (see Supplementary Material Figure S1).

This paper is the third in a series aiming to assess how x-CSI detector performance varies as a function of sensor thickness, pixel pitch, X-ray flux and CSCA choice. The complete data set analysed for these 3 papers comprised 3575 simulations, each assessed according to 4 different metrics. As a result of the many degrees of freedom considered, a detailed discussion of all data in a single paper would be unwieldy. The first paper identified the sensor thickness of interest as 1.5 mm (the value used in this work). The second paper identified trends in CSCA performance and explained these in terms of the different CSCA’s susceptibility to CSEs and pulse pileup. Relevant conclusions from those papers will be summarised and used to analyse the performance of the subtractive CSCAs considered in this paper, exploring how they differ from their additive counterparts. This paper considers CSCAs at all simulated pitches but only a single pixel thickness. Whilst the behaviours described here are consistent across all simulated fluxes, figures will only be included for two of the fluxes examined: the lowest and second highest fluxes (10^6^ and 10^8^ photons mm^−2^ s^−1^). These fluxes are sufficiently far apart to show how the behaviour of the various CSCAs change with increasing pulse pileup, without extending to the point where detector paralysis sets in for most CSCAs (which it has by 10^9^ photons mm^−2^ s^−1^, see Figure 4). For completeness, the full set of figures for all fluxes can be found in Supplementary Material Figure S1.

## 3. Results

It should be noted that x-CSI detector performance depends on a range of parameters other than CSCA choice, such as material purity, bias voltage, shaping time, etc. For this reason, this study does not attempt to predict absolute CSCA performance values but rather to identify and explain differences between trends in additive and subtractive CSCA performance. 

**Figure 5:**
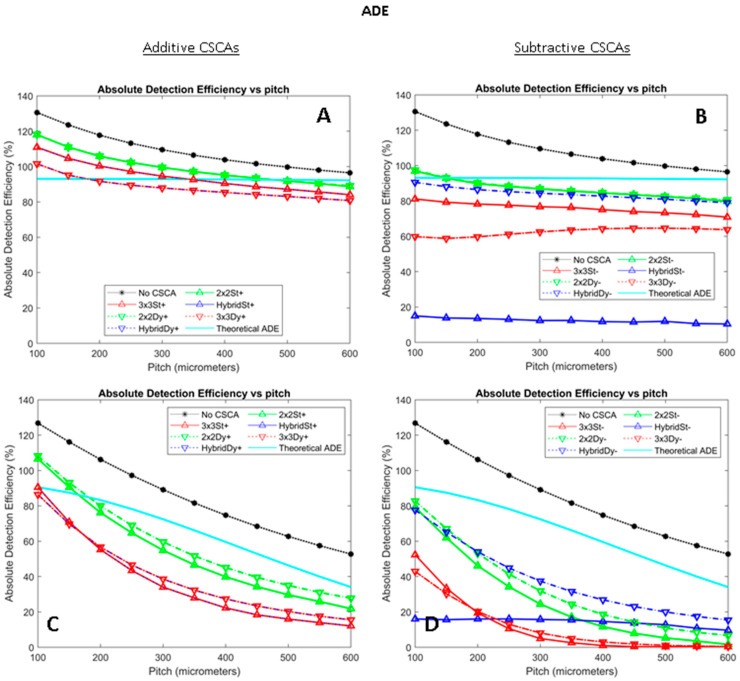
Plots showing how absolute detection efficiency (ADE) varies as a function of pixel pitch for additive (left column, (**A**,**C**)) and subtractive (right column, (**B**,**D**)) CSCAs at low (top row, (**A**,**B**)) and high (bottom row, (**C**,**D**)) X-ray fluxes. Sensor thickness was 1.5 mm in all cases. Low flux here refers to 10^6^ photons mm^−2^ s^−1^, and high flux refers to 10^8^ photons mm^−2^ s^−1^. Additive CSCA data reproduced from [22] for comparison with newly presented subtractive CSCA data.

Figure 5 shows how ADE varies for the simulated CSCAs as a function of pixel pitch and X-ray flux. For this metric, the performance of an idealised CdTe sensor, which does not experience any charge sharing, is also shown in light blue for reference. Comparing additive and subtractive approaches, various additive CSCAs produced a narrower range of ADE values than their subtractive counterparts, with the subtractive CSCAs also generally producing lower ADEs. In particular, additive 3 × 3 and Hybrid CSCAs performed almost identically, whilst the subtractive 3 × 3 and Hybrid CSCAs behaved markedly differently, with the subtractive dynamic Hybrid CSCA (HybridDy-) performing significantly better than the other 3 × 3 or Hybrid CSCAs at both high and low X-ray fluxes.

**Figure 6:**
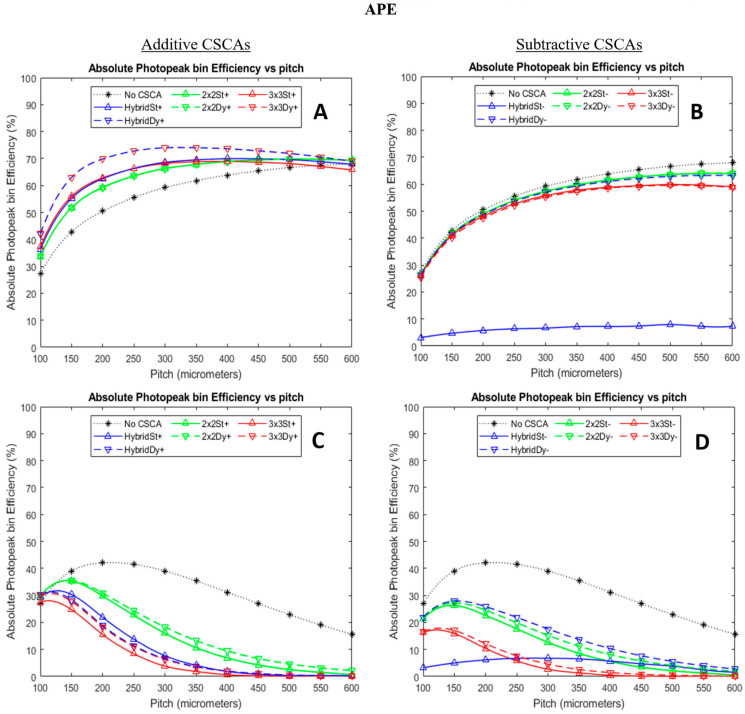
Plots showing how absolute photopeak efficiency (APE) varies as a function of pixel pitch for additive (left column, (**A**,**C**)) and subtractive (right column, (**B**,**D**)) CSCAs at low (top row, (**A**,**B**)) and high (bottom row, (**C**,**D**)) X-ray fluxes. Sensor thickness was 1.5 mm in all cases. Low flux here refers to 10^6^ photons mm^−2^ s^−1^, and high flux refers to 10^8^ photons mm^−2^ s^−1^. Additive CSCA data reproduced from [22] for comparison with newly presented subtractive CSCA data.

Figure 6 shows how APE varies for the simulated CSCAs as a function of pixel pitch and X-ray flux. An ideal APE score would be 100%. In contrast to ADE, subtractive CSCAs produce a comparatively narrower range of APE values than their additive counterparts at low X-ray fluxes (A and B), with the exception of the subtractive static Hybrid CSCA, which performed poorly under all measured conditions by this metric. 

**Figure 7:**
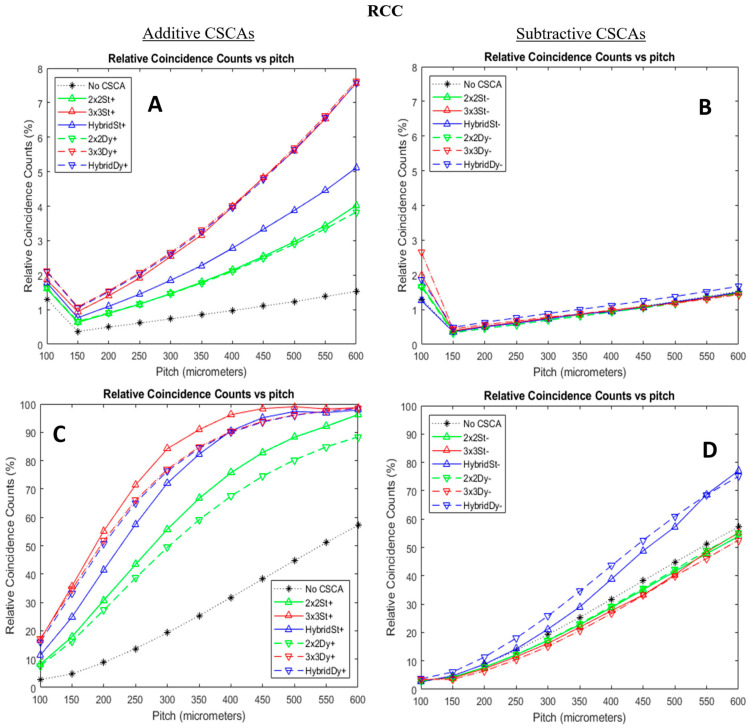
Plots showing how relative coincident counts (RCC) vary as functions of pixel pitch for additive (left column, ((**A**,**C**)) and subtractive (right column, (**B**,**D**)) CSCAs at low (top row, (**A**,**B**)) and high (bottom row, (**C**,**D**)) X-ray fluxes. Sensor thickness was 1.5 mm in all cases. Low flux here refers to 10^6^ photons mm^−2^ s^−1^, and high flux refers to 10^8^ photons mm^−2^ s^−1^. Additive CSCA data reproduced from [22] for comparison with newly presented subtractive CSCA data.

Figure 7 shows how RCC varies for the simulated CSCAs as a function of pixel pitch and X-ray flux. RCC ideally would record 0%. Subtractive CSCAs reduce RCC values recorded for almost all systems they are employed in, maintaining close to the No CSCA case at all but the highest per-pixel fluxes considered. This is the ideal behaviour that could be expected, given that pileup in the No CSCA case must be within a single pixel and, therefore, cannot be corrected for by a multi-pixel CSCA.

**Figure 8:**
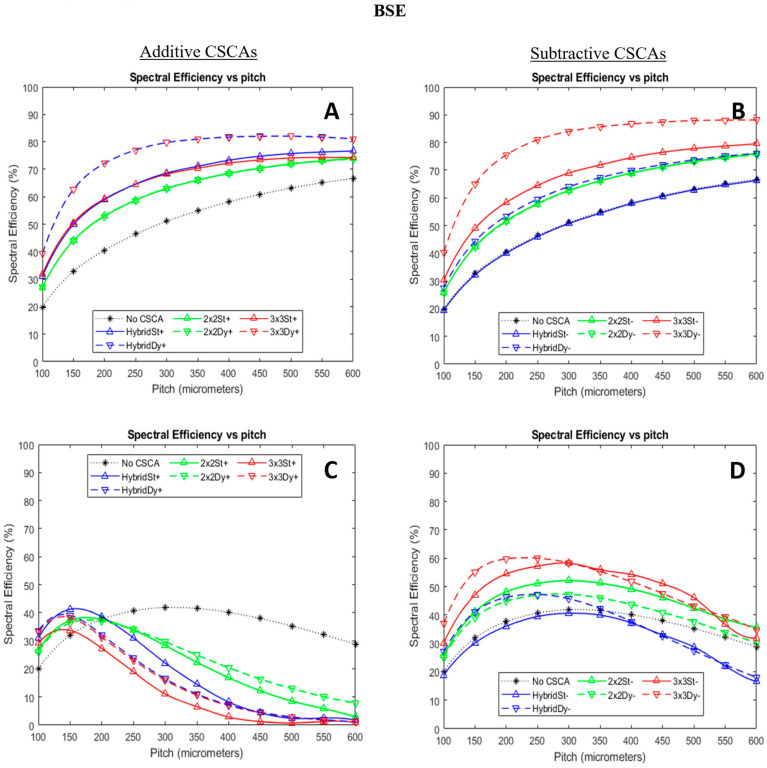
Plots showing how the binned spectral efficiency (BSE) varies as a function of pixel pitch for additive (left column, (**A**,**C**)) and subtractive (right column, (**B**,**D**)) CSCAs at low (top row, (**A**,**B**)) and high (bottom row, (**C**,**D**)) X-ray fluxes. Sensor thickness was 1.5 mm in all cases. Low flux here refers to 10^6^ photons mm^−2^ s^−1^, and high flux refers to 10^8^ photons mm^−2^ s^−1^. Additive CSCA data reproduced from [22] for comparison with newly presented subtractive CSCA data.

Figure 8 shows how BSE varies for the simulated CSCAs as a function of pixel pitch and X-ray flux. This metric would ideally be 100%. Subtractive CSCAs outperform their additive counterparts by this metric, producing higher peak efficiencies at low X-ray flux and dropping in performance more slowly as per-pixel X-ray flux increases. The slower drop in performance is so pronounced that even the subtractive static Hybrid CSCA, which performs worse than any additive CSCA at low flux, outperforms all additive CSCAs at the highest per-pixel X-ray fluxes simulated.

## 4. Discussion

The relative performance of the additive CSCAs is explained in terms of physical mechanisms in our previous publication [22], and the evidence for these mechanisms will not be repeated here. This is partly because the role of geometric effects will likely be reversed for subtractive algorithms compared with additive algorithms so that the factors that cause Geometric Advantage in additive CSCAs will produce Geometric Disadvantage in subtractive CSCAs, as explained in our previous work. The concept of Variable Pixelation introduced in that work holds equally well for both classes of CSCA but is of more use in comparing the relative performance of static and dynamic CSCAs than additive vs. subtractive CSCAs. For these reasons, this work instead explores how subtractive CSCAs compare with their additive counterparts in terms of the more basic mechanisms of pulse pileup and charge sharing effect. Differences between low and high flux responses of the subtractive CSCAs are similarly explained in these terms.

For most of the metrics assessed in this work, the best performance is the same as the highest or the lowest metric value (depending on the metric). For ADEs, however, this is not the case as CSCAs can either over- or under-correct for charge sharing, resulting in ADEs being pushed either higher or lower. To assess CSCA performance according to this metric, an analytical model was developed to show the behaviour of an idealised sensor with no charge sharing, as detailed in Section 2.7. The results of this model are shown in cyan (light blue) in Figure 5.

Figure 5 shows that as the X-ray flux increases from 10^6^ to 10^8^ photons mm^−2^ s^−1^, additive CSCAs fall from being primarily above the idealised ADE line to primarily below it (at all but the lowest pixel pitches). This shift indicates a change in the dominant form of distortion in the system, moving from charge sharing effects (which increase ADE) to pulse pileup (which lowers ADE). In contrast, the subtractive CSCAs were almost exclusively below the idealised ADE at all X-ray fluxes considered, indicating their lower susceptibility to charge sharing effects, as designed. The subtractive CSCAs also demonstrate a notably broader range of performances than their additive counterparts. In particular, there are three results which need commenting on:

(1) Hybrid and 3 × 3 CSCAs are indistinguishable in the additive case but markedly different in the subtractive case;
(2) There is a large increase in the difference between static and dynamic CSCAs in all but the 2 × 2 case (when comparing additive and subtractive CSCAs);
(3) In the subtractive case only, the performance of static vs. dynamic versions of the Hybrid CSCAs is the reverse of the 3 × 3 case.

These differences can be explained by comparing the Hybrid and 3 × 3 CSCAs in both additive and subtractive forms, specifically with respect to ADE. ADE only considers whether a count is incremented, without reference to its energy. In the case of additive CSCAs, Hybrid and 3 × 3 will both output a single count from a 3 × 3 area (even though they may differ in energy), so they score identically for ADE. Similarly, the difference between static and dynamic CSCAs in additive mode is simply a function of how likely the shared charge is to lie within the same search area, as explained in our previous publication on additive CSCAs [22], which leads to quite a low disparity between static and dynamic approaches for larger search areas. 

**Figure 9:**
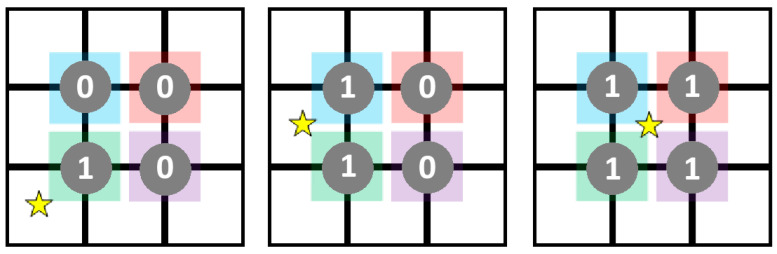
The 3 × 3 search areas used for a subtractive Hybrid CSCA are shown. The charge from a detected photon is shown as a yellow star, and the four possible 2 × 2 sub-regions are shown by coloured squares. The total charge assessed by each subregion is shown as a white number in a grey circle. If only a single event occurs, and it occurs in a corner or edge pixel ((**left**) and (**centre**) figures, respectively), then at least one 2 × 2 sub-region exists with no charge in it, meaning that the output will be 0, and no count will occur. If the photon is detected in the centre pixel however ((**right**) figure), then all 2 × 2 sub-regions will contain it, meaning that a non-zero output (1 in this case) is guaranteed from the CSCA. This explains the lower number of counts recorded for absolute metrics that use static vs. dynamic subtractive CSCAs.

In the subtractive case, however, whilst the 3 × 3 will simply not count if it detects any form of charge sharing within its search area (two or more pixels activate), the Hybrid CSCA may still output a count if it cannot identify a 2 × 2 region within its search area that would score below threshold (see Figure 9 below). This reasoning also explains why the static Hybrid produces so many fewer counts than the dynamic Hybrid in subtractive mode: by ensuring that one of the events is in the centre of the search area, the dynamic Hybrid ensures that ADE is incremented for every event detected, whilst the static Hybrid is almost always able to find a 2 × 2 region which scores below threshold and so will not output a count, as long as no event is detected by the centre pixel. This difference in behaviour also explains the third of the major differences noted: centring on a charge sharing event decreases the probability that a 3 × 3 CSCA will record a count (by maximising the chance of detecting charge sharing and suppressing the count), whilst the same centring increases the probability that the Hybrid CSCA will record a count (by ensuring the lowest possible 2 × 2 region is above the counting threshold).

Turning to Figure 6, there are similar differences between additive and subtractive CSCAs, with the notably low performance of the subtractive Hybrid static CSCA and the increased difference between Hybrid and 3 × 3 CSCAs in the subtractive case. This is to be expected as this is another absolute metric, so the same arguments regarding how the static vs. dynamic approach affects a count being recorded still apply. However, there are notable additional differences in this metric, including that all subtractive CSCAs perform more poorly at lower fluxes, but the subtractive Hybrid CSCAs perform better than their additive counterparts at higher fluxes (at pitches > 200 µm for the dynamic and >350 µm for the static cases). At the higher fluxes considered here, it is also notable that the dynamic Hybrid CSCA outperforms even the 2 × 2 CSCAs in the subtractive case, in contrast to the additive case where the 2 × 2 is the top performing CSCA.

These differences demonstrate the main advantage of subtractive CSCAs over additive ones, which is that they are much better at suppressing spectral distortions caused by charge sharing and pulse pileup. Under low flux conditions, this is not immediately evident as the performance of the subtractive CSCAs is inferior to the additive ones. This is because APE is an absolute metric so is limited by the total number of counts recorded by the detector. At lower fluxes where pileup is minimal, the benefit of subtractive approaches in excluding pileup and charge sharing is not sufficient to offset the cost of discarding events that involve any charge sharing. As a result, additive approaches, which will always produce a count even in a charge sharing case, perform better. At higher fluxes, it can be seen that the subtractive approaches drop off in performance far more gradually than the additive versions, performing better across a wide range of pixel pitches. This is because the increasing pixel pitch leads to an increase in pulse pileup (which affects additive CSCAs more strongly) and, simultaneously, a reduction in charge sharing (which affects the APE in subtractive CSCAs more severely). In general, however, the APE of a subtractive CSCA is worse than its additive counterpart, even at these high fluxes, as again, APE is an absolute metric, so any counts thrown away will affect it.

The one glaring exception to this rule is the Hybrid CSCAs, and the reasons for this again come down to the fact that the subtractive Hybrid CSCAs do not always suppress counts and may, under some circumstances, produce a count. In particular, as the CSCAs read out the lowest 2 × 2 grouping of pixels in their 3 × 3 search area, subtractive Hybrid CSCAs under high flux will favour reporting the result of a corrected charge sharing event over a pileup event, as the pileup of two unrelated photons will statistically result in a higher energy than the reconstruction of a single photon, split by charge sharing. This ability to ignore a pileup in favour of reconstructing a charge sharing event is unique to the subtractive Hybrid CSCAs, explaining their superior performance at the higher X-ray fluxes, even over the 2 × 2 CSCAs. In fact, this effect can be so significant that the subtractive static Hybrid CSCA goes from being the worst-performing CSCA to one of the best at high fluxes and large pixels, specifically because its performance appears so robust to noise (though not particularly high in absolute terms).

Turning to the difference between the static and dynamic versions of these two algorithms at high flux, the dynamic CSCA performs better as by centring the 3 × 3 search area around one detected event, it makes it impossible to find a 2 × 2 subregion containing no events and, thus, does not suppress counting in the same way that the static Hybrid can. Again, this applies as APE is an absolute metric, so any count suppression reduces performance.

In contrast to the absolute metrics of APE and ADE, Figure 7 shows how the various CSCAs score according to their RCC, which is a relativistic metric. The subtractive CSCAs are designed to prevent spectral distortions by not outputting counts when pixels activations are close in space and time. Whilst this is designed to prevent charge sharing events from degrading the spectrum, Figure 7 shows that it also has the effect of suppressing pileup counts. As a result, all subtractive 2 × 2 and subtractive 3 × 3 CSCAs produce less pileup than even the No CSCA case. This is in stark contrast to the additive CSCAs, which all vastly increase pileup, even at low flux. There are three behaviours to note about the subtractive CSCAs in these figures in particular:

(1) the best-performing CSCAs by absolute metrics (Hybrid) are the worst-performing by RCC (they have the highest RCCs);
(2) the difference between static and dynamic Hybrid CSCAs is less marked in RCC than in APE or ADE;
(3) at low fluxes and the smallest pixel pitches, the ordering of the CSCAs is reversed, with almost all performing worse than the No CSCA case.

**Figure 10:**
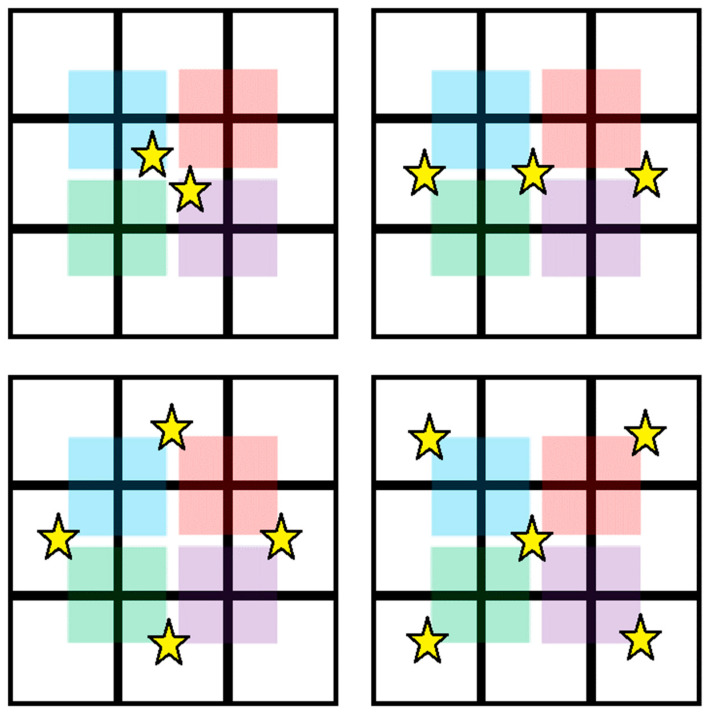
Subtractive Hybrid CSCAs select the lowest possible charge from the four possible 2 × 2 sub-regions within their search area. For two photons to trigger a pileup count, both need to be in the centre pixel (**top left**). For only three photons (**top right**), it requires a line running through the centre pixel. Avoiding the centre pixel would require at least four photons (**bottom left**), and avoiding edge pixels would require five (**bottom right**).

The first of these phenomena is easy to explain in light of the earlier discussions. Most subtractive CSCAs can only record RCC when there is a true pileup event (two unrelated photons arrive at the same time) in a single pixel. The subtractive Hybrid CSCAs, however, act somewhere between the strictly reconstructive and strictly subtractive 3 × 3 CSCAs, sometimes outputting no signal but occasionally outputting the lowest possible charge. Figure 10 shows how as few as three photons simultaneously incident on a 3 × 3 area can result in a pileup count being recorded by a subtractive Hybrid CSCA, where a subtractive 3 × 3 or even 2 × 2 would suppress the count. This figure also explains the closer performance between Hybrid and Static CSCAs by this metric. RCC records pileup, which in a subtractive Hybrid CSCA requires multiple photons in the same 3 × 3 area. The way to do this with the fewest photons involves using the middle pixel and two edge pixels. Whilst this can be achieved with four or five photons, the high improbability of so many unrelated photons interacting in such an organised way within such a small area means that, in reality, a pileup count in a subtractive Hybrid CSCA is likely associated with either two or three photons, as shown in Figure 10. As two and three-photon pileups require centre pixel involvement to record a count, there is a slightly higher chance of observing these pileup counts in the dynamic version. The RCC count is still largely governed by the statistical distribution of coincident photons within the 3 × 3 area, however, so the difference between the dynamic and static cases is less pronounced than in the absolute metrics, which only require that a count of any energy is recorded. 

Combined, the above arguments explain why the subtractive Hybrid CSCAs perform intermittently between the worst-performing additive and best-performing subtractive CSCAs. Notably, though they suffer from pileup more than No CSCA case (unlike the other subtractive CSCAs, which all have lower RCC values), the subtractive Hybrid CSCAs are affected by pileup far less than any additive CSCA, most of which saturate at near 100% RCC at this X-ray flux (10^8^ photons mm^−2^ s^−1^).

The final feature of note from this figure is that the ordering of the subtractive CSCAs by performance at the smallest pixel size is reversed compared with all other pixel sizes (at 100 µm the 3 × 3 dynamic is worst and Hybrid dynamic best, whilst at 150 µm and greater the 3 × 3 dynamic is the best, and Hybrid dynamic is the worst). This effect is not seen in the additive CSCAs. To understand why this is, we note the following:

- Charge sharing effects are only dominant at the smallest pixel pitches and lowest fluxes;
- At larger pixel pitches and X-ray fluxes, pulse pileup is the dominant distortion effect;
- Pileup increases RCC for both additive and subtractive CSCAs;
- Correcting for charge sharing reduces the counts recorded by subtractive CSCAs but does not affect the counts recorded for additive CSCAs (which still score 1 count);
- Removing non-pileup counts from the output will increase RCC, as true coincidence counts (those in the same pixel) will represent a higher proportion of the output counts.

From this, we can reason that the subtractive CSCAs show an inversion in their ordering when there is a transition from a pileup-dominant to a charge sharing dominant regime. This is because subtractive CSCAs, which are best at identifying pileup (reducing RCC), will also be best at identifying charge sharing (increasing RCC).

Finally, Figure 8 shows how the spectral efficiency of the different CSCAs varies with pixel pitch at lower and higher X-ray fluxes. Spectral efficiency is an important metric for X-ray photon-counting detectors as it represents how well the detector is able to accurately assess the energy of an incoming photon. This metric can, thus, be used as a proxy for how well the spectral information, which is vital for material decomposition and quantification tasks, is preserved by any given CSCA. The first thing to note is that the neighbourhood sizes considered are all affected differently by the move from additive to subtractive CSCA: the 2 × 2 CSCAs behave roughly the same; the 3 × 3 CSCAs perform markedly better in the subtractive case, whilst the Hybrid CSCAs perform markedly worse in subtractive form. This can be understood by considering the different ways additive and subtractive CSCAs work and, particularly, how pulse pileup can interfere with them. Additive CSCAs work by identifying a group of closely related events and attempting to reconstruct the original signal, but this approach assumes that the closely related events are actually from charge sharing and not pulse pileup. At low X-ray fluxes and small pixel sizes, this assumption is reasonable, and the reconstruction is mostly successful, making additive approaches superior to their subtractive counterparts. This is, thus, the case for the 2 × 2 CSCAs. As the search area increases, however, so too does the probability that events found correlated in time will be the result of pileup rather than charge sharing, and this is why, for 3 × 3 CSCAs, the subtractive versions perform better. As previously mentioned, Hybrid CSCAs search through an area of 3 × 3 but then report from a smaller 2 × 2 region, and so again, their change in performance is intermediate between the other two cases.

At higher flux, the picture is much more straightforward, with subtractive CSCAs outperforming their additive counterparts at all, except the lowest pixel pitches (where charge sharing effects again become significant compared with pule pileup, so additive CSCAs have the advantage). Notably, and in stark contrast to the additive CSCAs, subtractive CSCAs are able to significantly improve spectral efficiency compared with the No CSCA case at high flux, with the exception of the Hybrid CSCAs that, again, perform intermediately between additive and subtractive CSCAs, as discussed in the RCC section. The ordering of subtractive CSCAs remains the same at high flux as at low flux: 3 × 3 subtractive CSCAs perform the best, followed by 2 × 2 and then Hybrid.

## 5. Conclusions 

This work looked to compare subtractive CSCAs with their additive counterparts. Though designed specifically to reduce the occurrences of charge sharing in the output energy spectrum, subtractive CSCAs also had a significant effect on reducing pulse pileup within the output. These advantages came at a significant cost, however, with subtractive CSCAs performing progressively poorer on absolute metrics as X-ray fluxes increased due to the high number of counts they rejected. In a physical system, this correlates to a reduction in the sensitivity of the scanner, which may have consequences for image quality and dose efficiency in medical applications.

Offsetting these drawbacks are the significant advantages that subtractive CSCAs provide in terms of preserving spectral information. This can be seen most clearly at high fluxes, where subtractive CSCAs preserve significantly more spectral information than the No CSCA case or any additive CSCAs. For some medical applications, such as material differentiation tasks or contrast agent quantification, spectral information is vital, so trading off dose efficiency for spectral fidelity may be worth it.

Geometric considerations affect additive and subtractive CSCAs in opposite ways, whilst variable pixelation acts similarly on both. As a result, the behaviour of subtractive CSCAs is not easy to predict based solely on their additive counterparts. Hybrid CSCAs, in particular, show significant differences based on whether dynamic or static correction mechanisms are employed. Table 4 below summarises which CSCA class performs better according to whether dose considerations (counting efficiency) or spectral fidelity (spectral efficiency) is the more important consideration.

In conclusion, the choice of which correction approach to use with a CSCA will largely be based on the task at hand and will depend on the relative importance of spectral information and dose efficiency. This work provides guidance to allow for an informed selection of CSCA, extending our previous work establishing parameters of neighbourhood size and neighbourhood localisation to include a correction mechanism.

## Figures and Tables

**Table 1:** Simulation times used for each X-ray flux to ensure a consistent number of simulated photons.

X-ray Flux (Photons mm^−2^ s^−1^)	Simulated Time (ms)
10^6^	10
10^7^	1
10^8^	0.1
10^9^	0.01

**Table 2:** Material Properties for the simulated CdTe.

Parameter	Symbol	Value	Unit
Sensor thickness	T	1.5	mm
Mobility, electrons	µ_e_	1100	cm^2^ V^−1^ s^−1^
Mobility, holes	µ_h_	100	cm^2^ V^−1^ s^−1^
Lifetime, electrons	τ_e_	3.0	µs
Lifetime, holes	τ_h_	2.0	µs
Density	Ρ	5850	kg m^−3^
Diffusion coefficient, electrons	D_e_	2.84 × 10^−3^	m^2^ s^−1^
Diffusion coefficient, holes	D_h_	2.58 × 10^−4^	m^2^ s^−1^
Relative permittivity	ε	11.0	-

**Table 3:** Energy thresholds, energy bin locations and spectral features identified.

Bin Number	Threshold (keV)	Bin Range (keV)	Spectral Feature Enclosed
1	10	10–30	Fluorescence X-rays
2	30	30–60	Escape peak
3	60	60–83	Full-energy photopeak
4	83	>83	Pileup events

**Table 4:** Summary of which correction mechanism, additive or subtractive, is superior for each CSCA Neighbourhood Size depending on whether counting or spectral efficiency is prioritised. Results are shown for low and high X-ray fluxes.

		Best CSCA Correction Type		
	Prioritised Efficiency	2 × 2	3 × 3	Hybrid
**Low Flux**	Counting	Additive	Additive	Additive
	Spectral	Additive	Subtractive	Additive
**High Flux**	Counting	Additive	Additive	Additive if dynamic and <350 µm pitch; otherwise, subtractive
	Spectral	Subtractive	Subtractive	Subtractive

## Data Availability

The data presented in this study are available on request from the corresponding author.

## Supplementary Materials

The following supporting information can be downloaded at: https://www.mdpi.com/article/10.3390/s24154946/s1, Supplementary materials are available which include plots showing how APE, ADE, BSE, RCC, ‘total counts in coincidence bin’ and ‘total counts in photopeak bin’ vary as a function of pixel pitch for all X-ray fluxes simulated in this work.
