# Supplementary material for: To Reconstruct or Discard: A Comparison of Additive and Subtractive Charge Sharing Correction Algorithms at High and Low X-ray Fluxes
Source: Sensors (Basel). 2024 Jul 30;24(15):4946. doi: 10.3390/s24154946 (PMC11314781; doi:10.3390/s24154946)
Supplement: Supplementary file 1 [file sensors-24-04946-s001.zip › sensors-3100885-supplementary.pdf]

To Reconstruct or Discard: A Comparison of Additive and Subtractive Charge Sharing Correction Algorithms at High and Low X-Ray Fluxes

Oliver L. P. Pickford Scienti * and Dimitra G. Darambara

Joint Department of Physics, Institute of Cancer Research and Royal Marsden NHS Foundation Trust, London SM2 5NG, UK; dimitra.darambara@icr.ac.uk

* Correspondence: olie.scienti@icr.ac.uk

ADE

Flux	Additive	Subtractive
10^6 photons mm^{-2} s^{-1}	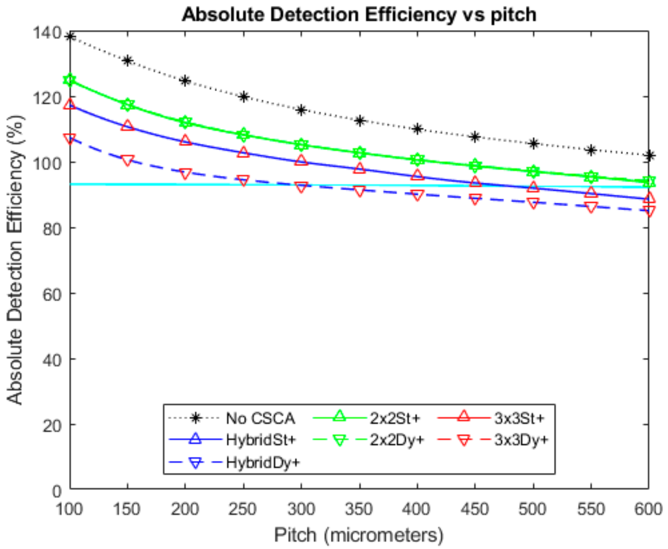	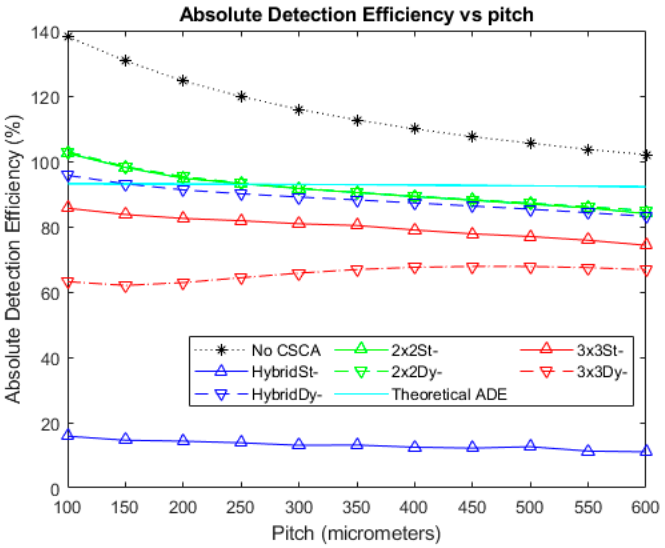

10^7 photons mm^{-2} s^{-1}	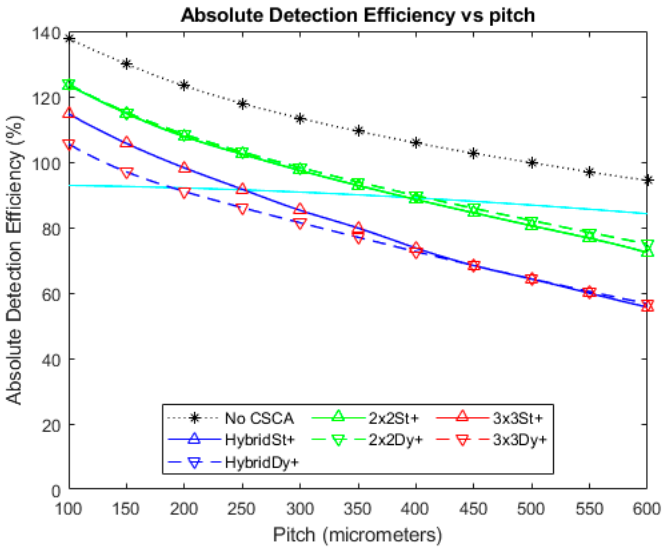	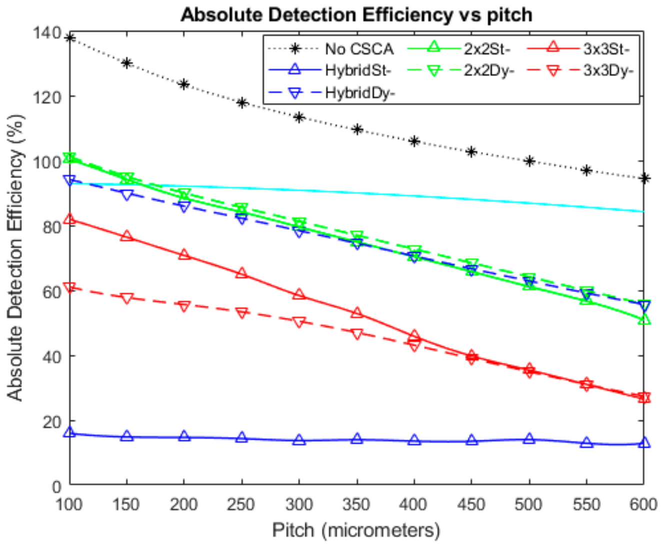
10^8 photons mm^{-2} s^{-1}	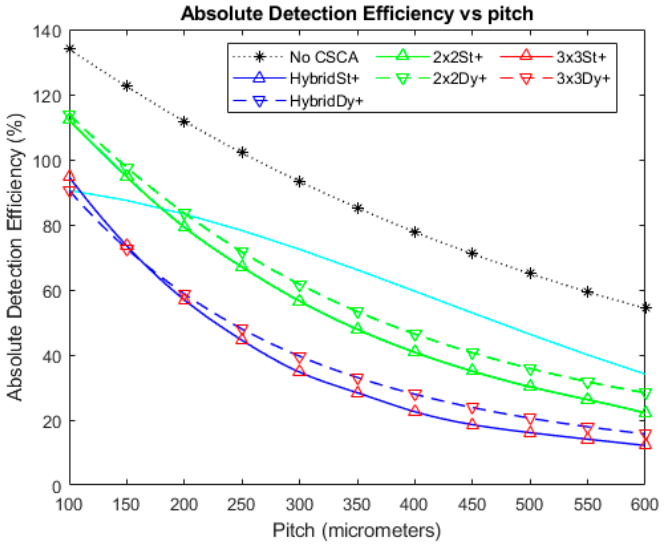	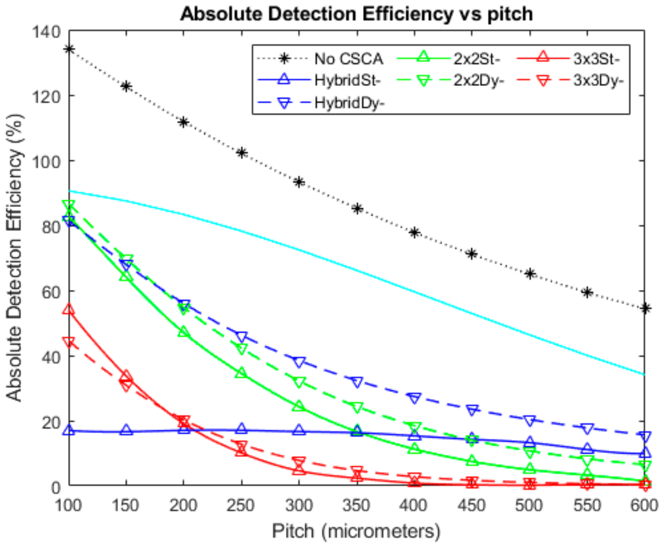

10^9 photons mm^{-2} s^{-1}	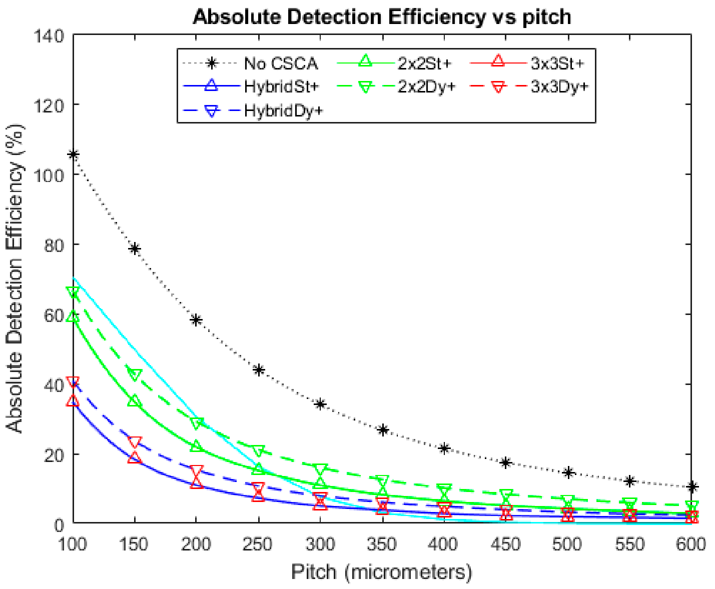	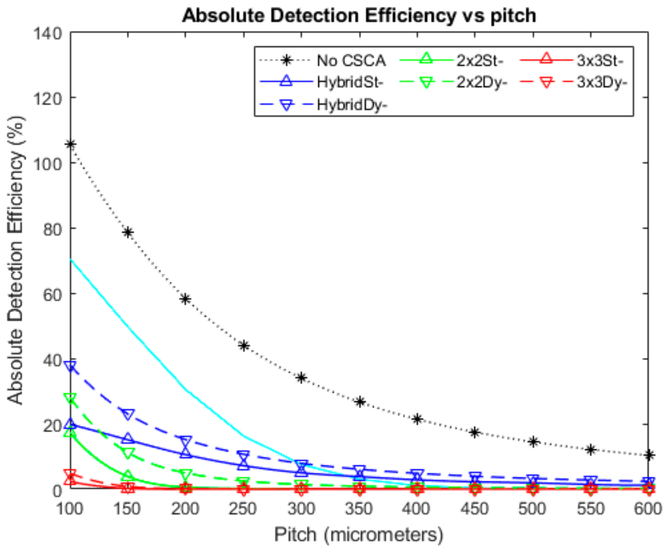

APE

Flux	Add	Sub
10^6 photons mm^{-2} s^{-1}	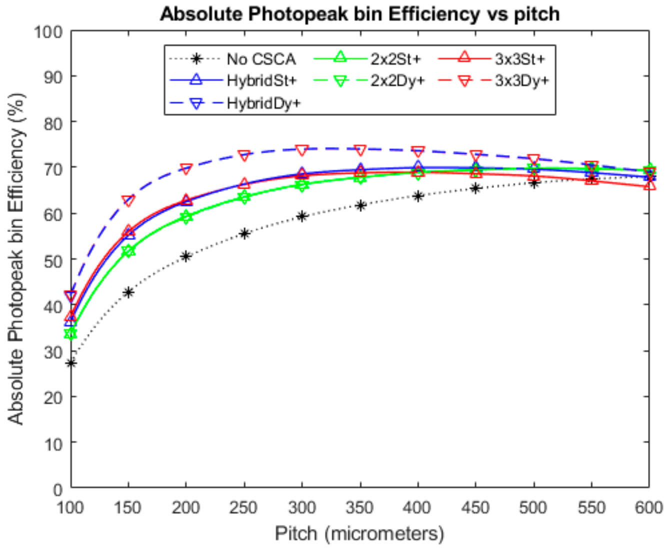	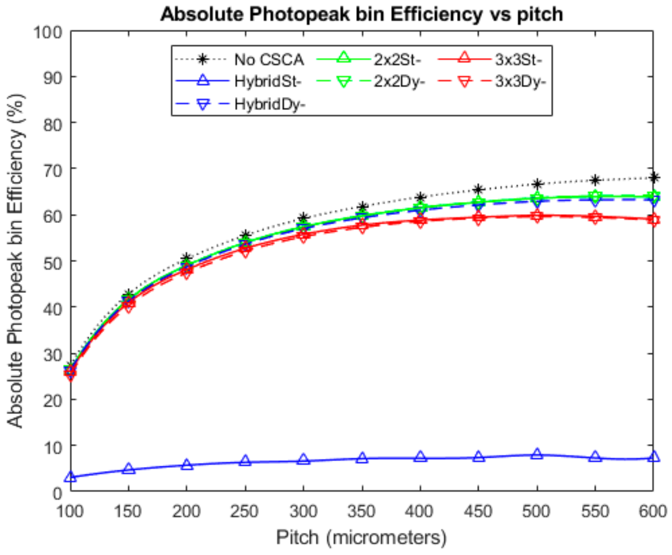

10^7 photons mm^{-2} s^{-1}	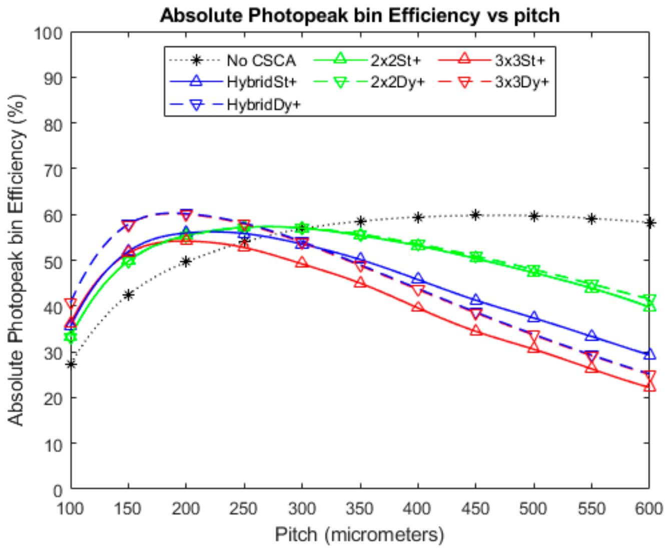	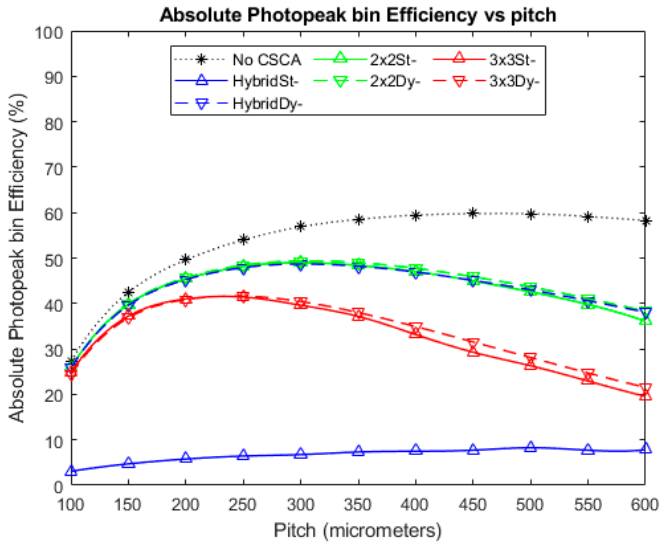
10^8 photons mm^{-2} s^{-1}	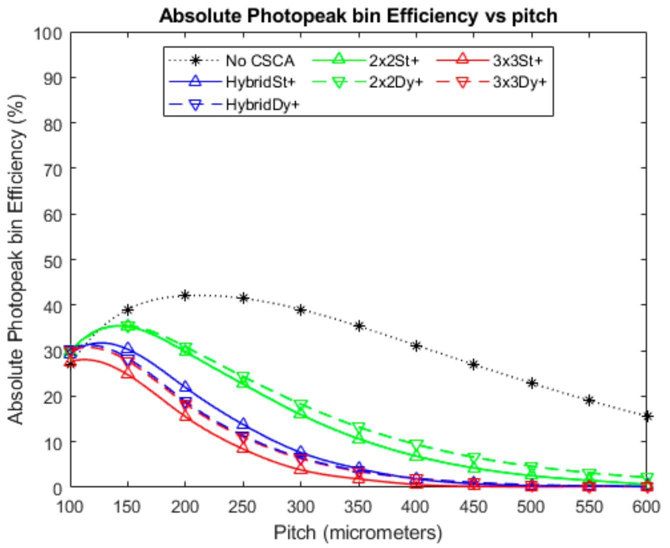	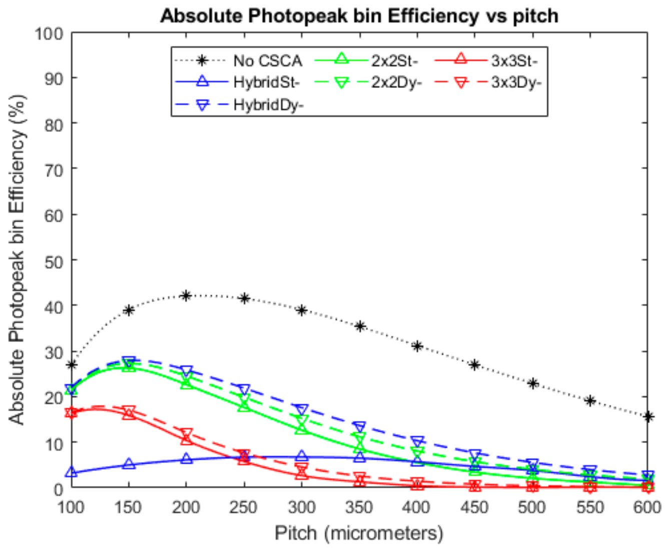

<table>
<tr><td>10^9 photons mm^{-2} s^{-1}</td><td>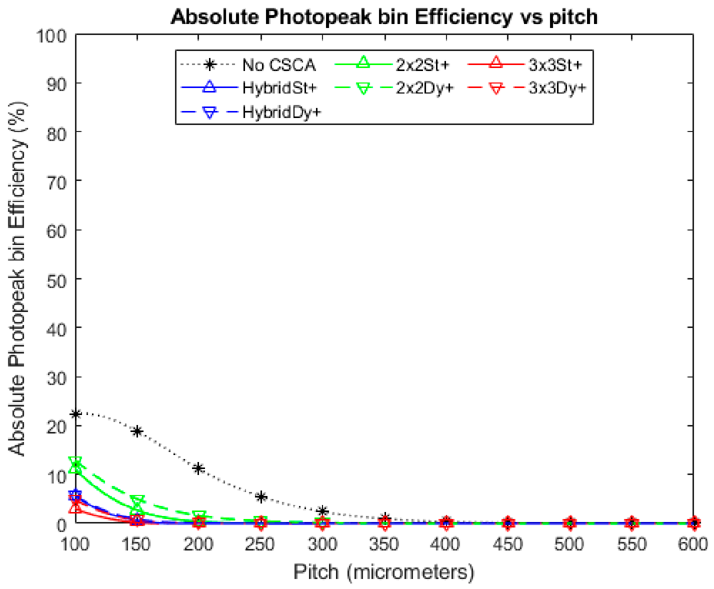
</td><td>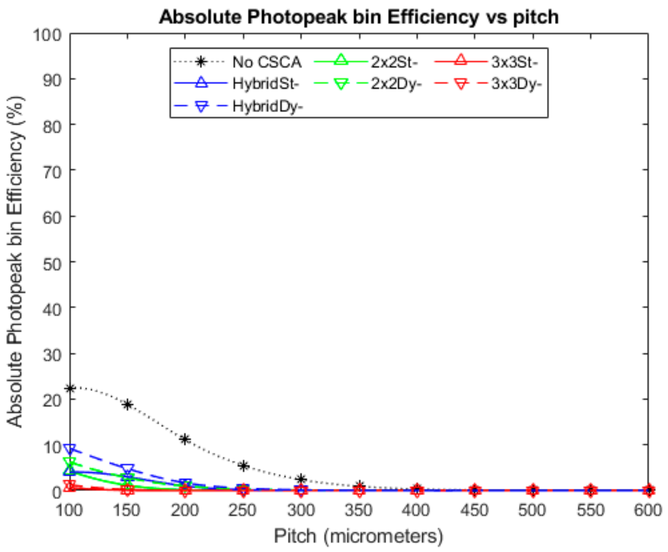
</td></tr>
</table>

RCC

<table>
<tr><th>Flux</th><th>Add</th><th>Sub</th></tr>
<tr><td>10^6 photons mm^{-2} s^{-1}</td><td>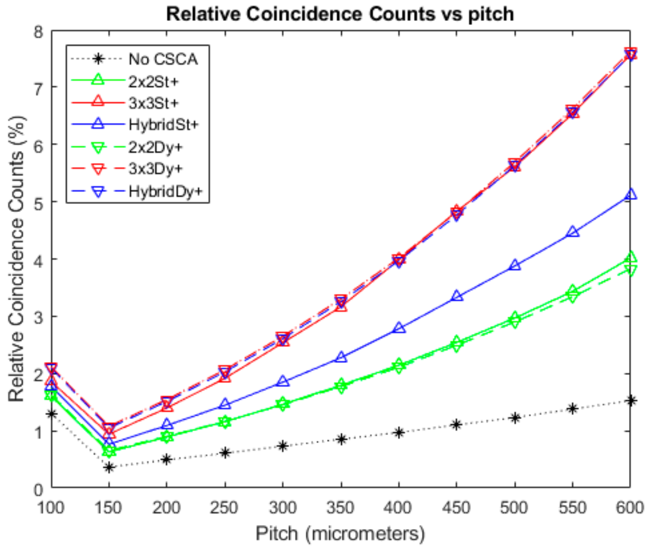
</td><td>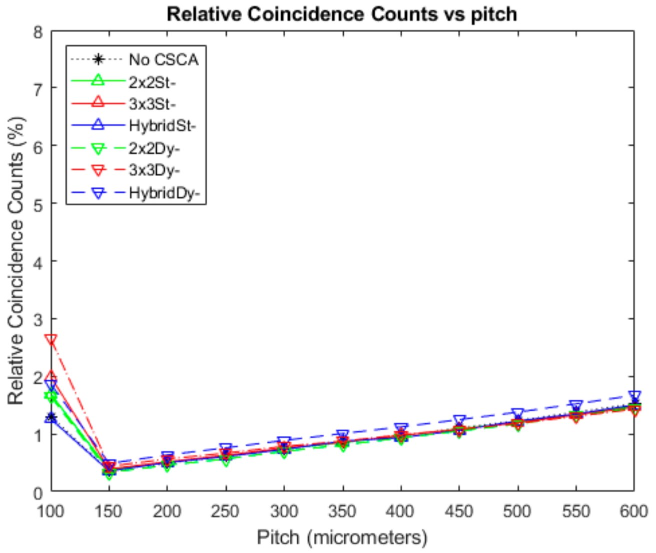
</td></tr>
</table>

<table>
<tr><td></td><td></td><td></td></tr>
<tr><td>10^7 photons mm^{-2} s^{-1}</td><td>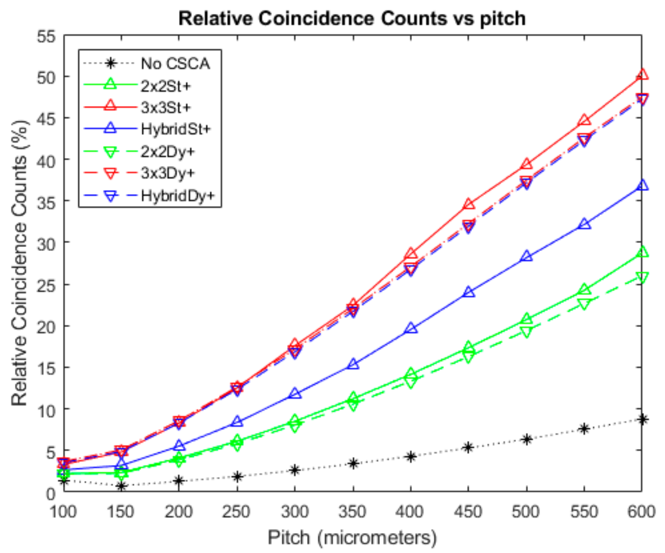
</td><td>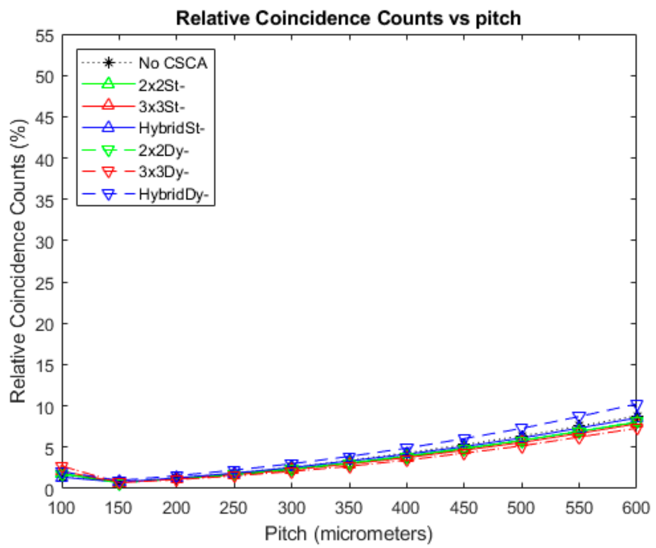
</td></tr>
<tr><td>10^8 photons mm^{-2} s^{-1}</td><td>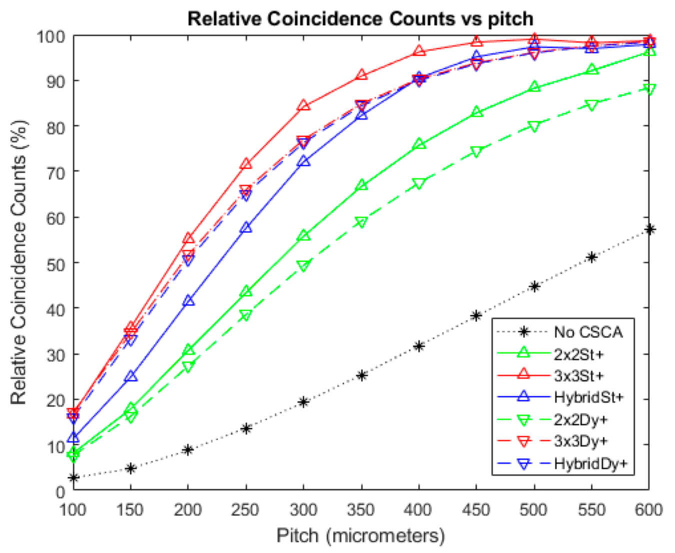
</td><td>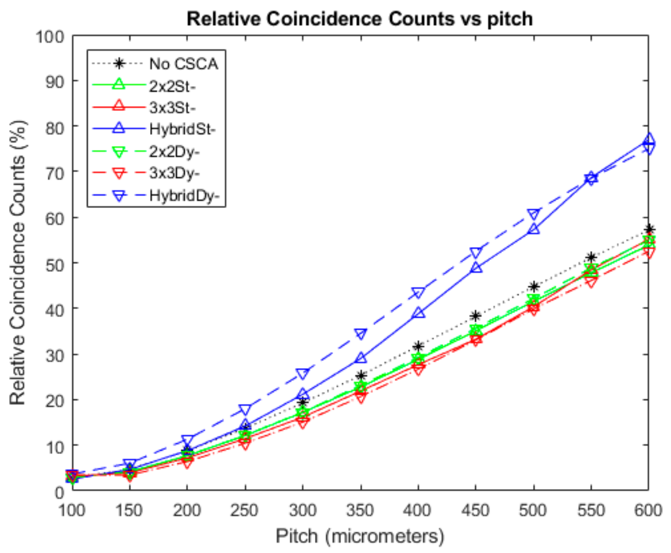
</td></tr>
</table>

<table>
<tr>
<td>10^9 photons mm^{-2} s^{-1}</td>
<td>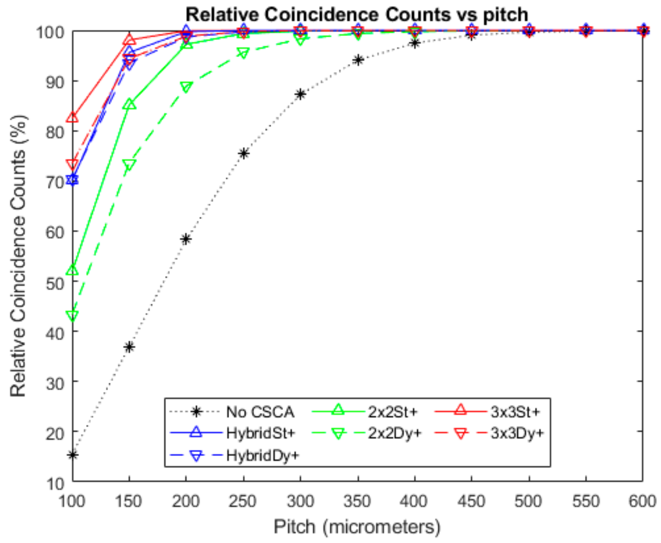</td>
<td>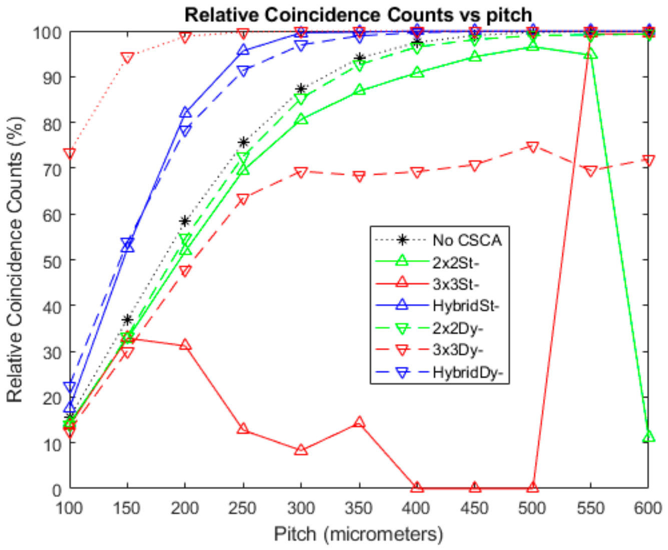</td>
</tr>
</table>

<u>Total counts in coincidence bin</u>

Flux	Add	Sub

<table>
<tr>
<td>10^6 photons mm^{-2} s^{-1}</td>
<td>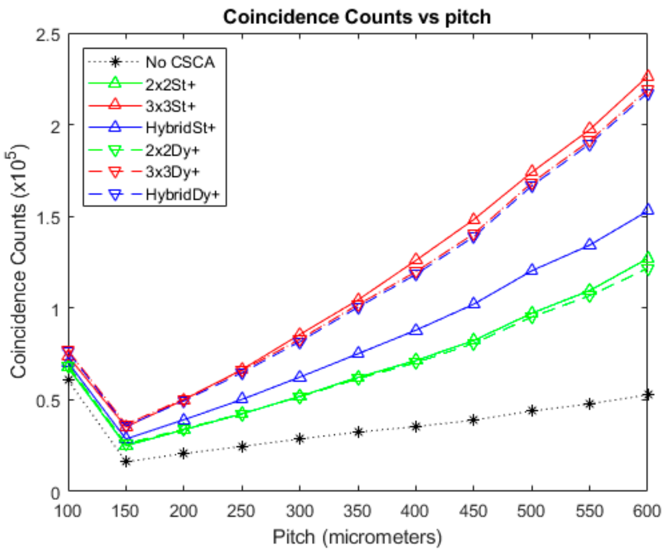
</td>
<td>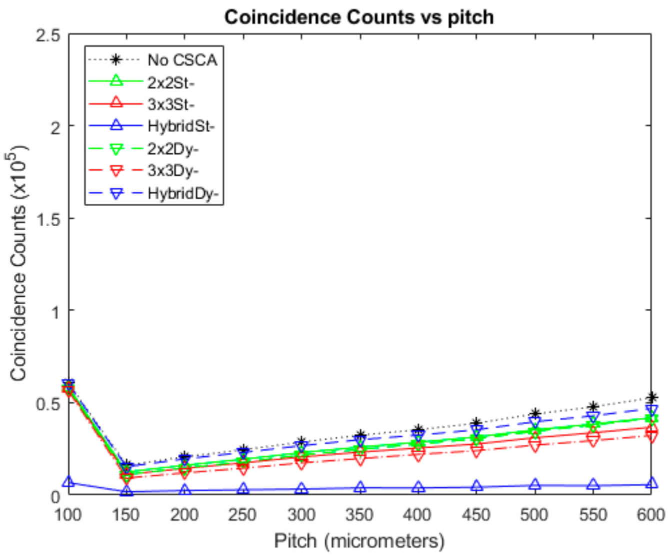
</td>
</tr>
<tr>
<td>10^7 photons mm^{-2} s^{-1}</td>
<td>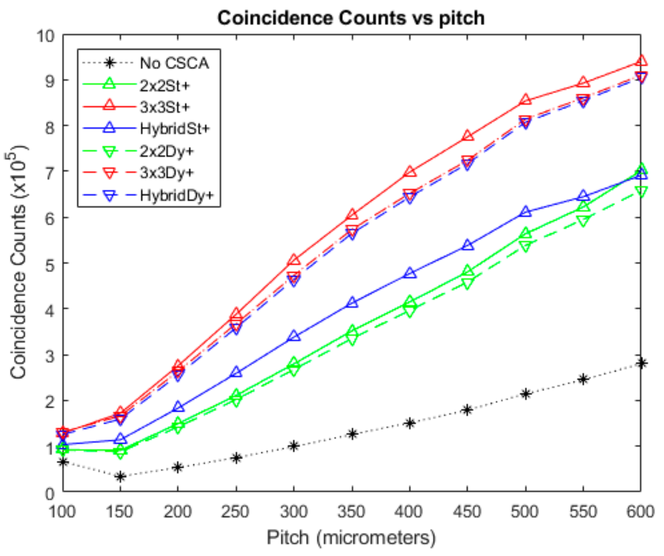
</td>
<td>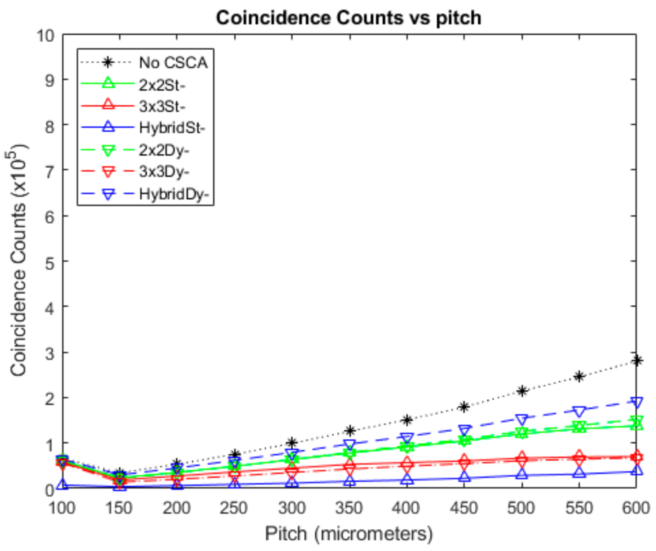
</td>
</tr>
</table>

<table>
<tr><td>10^8 photons mm^{-2} s^{-1}</td><td>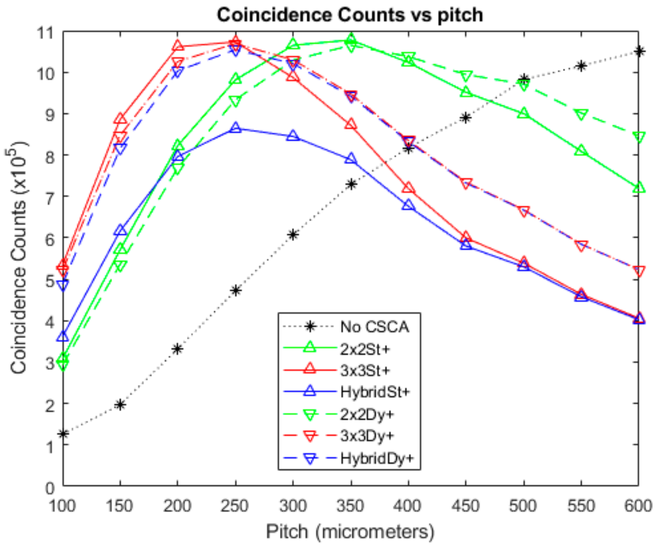
</td><td>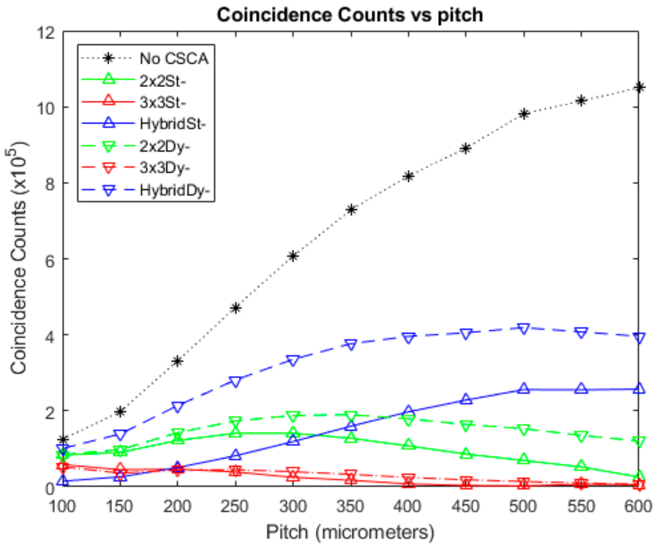
</td></tr>
<tr><td>10^9 photons mm^{-2} s^{-1}</td><td>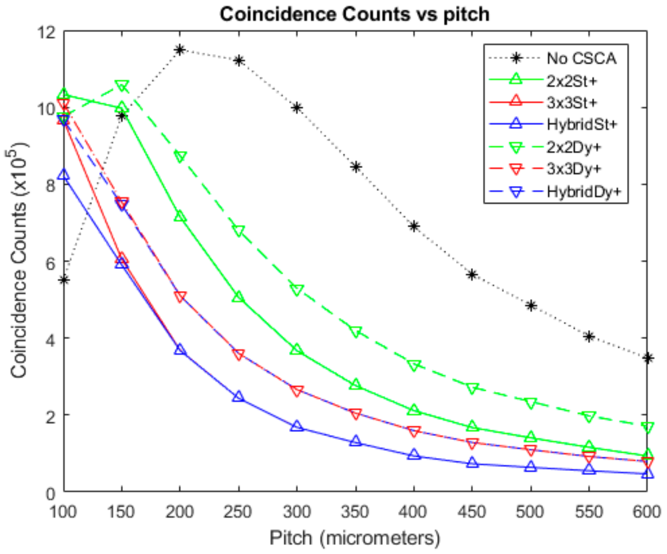
</td><td>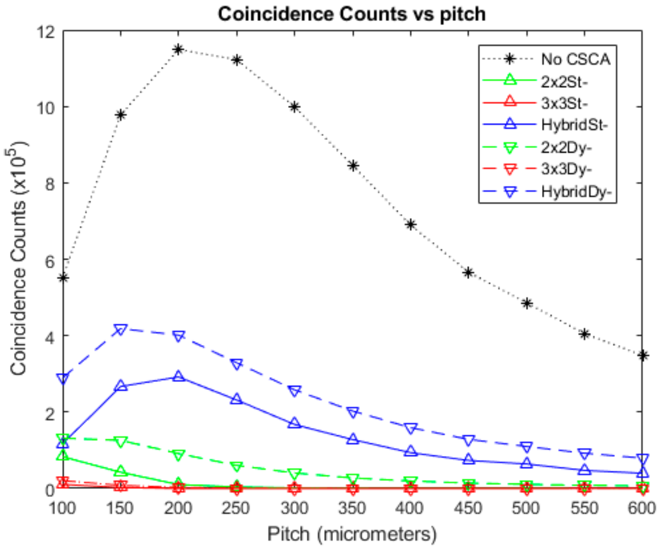
</td></tr>
</table>

BSE

Flux	Add	Sub

10^6 photons mm^{-2} s^{-1}	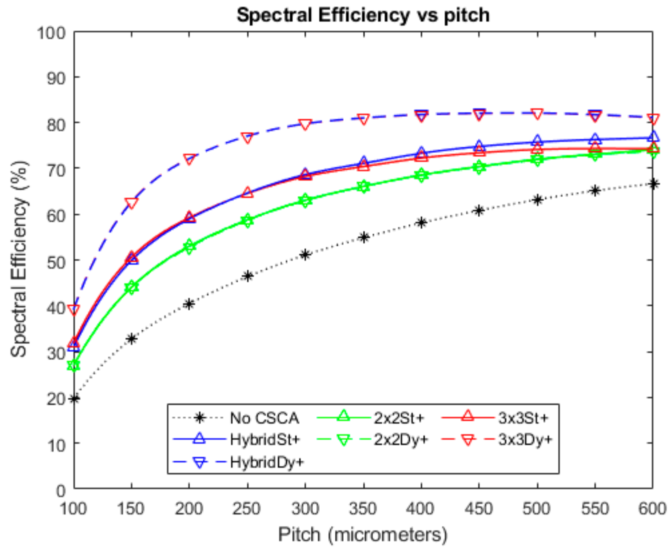	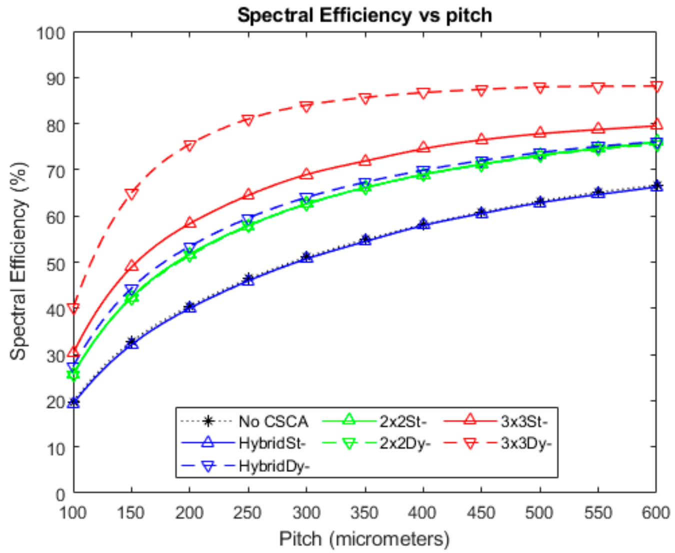
10^7 photons mm^{-2} s^{-1}	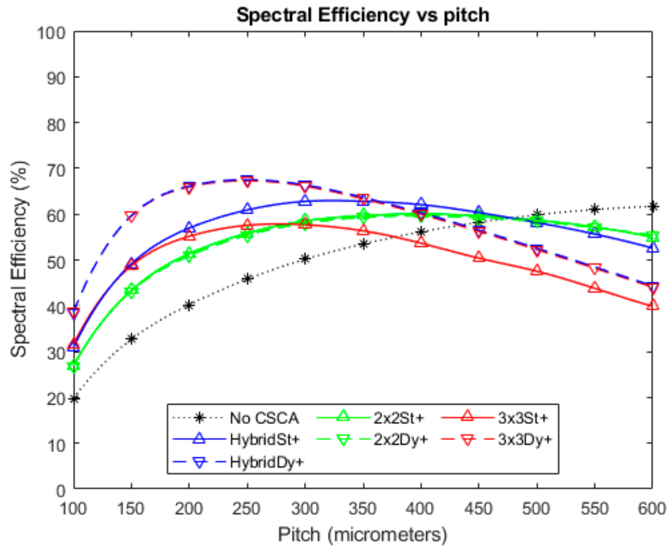	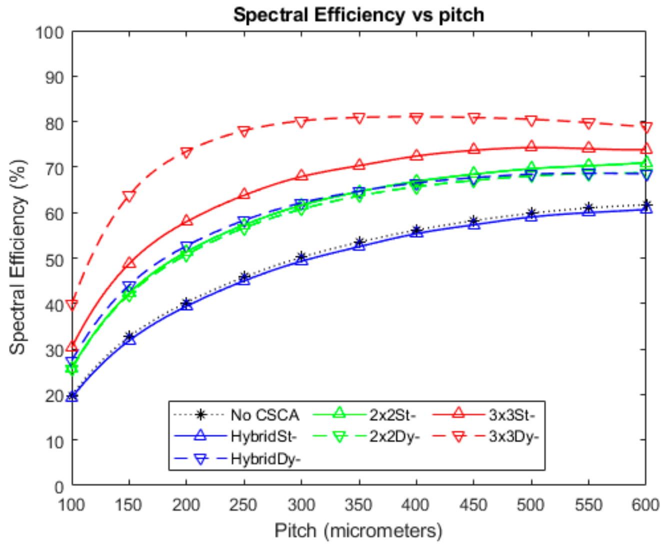

<table>
<tr><td>10^8 photons mm^{-2} s^{-1}</td><td>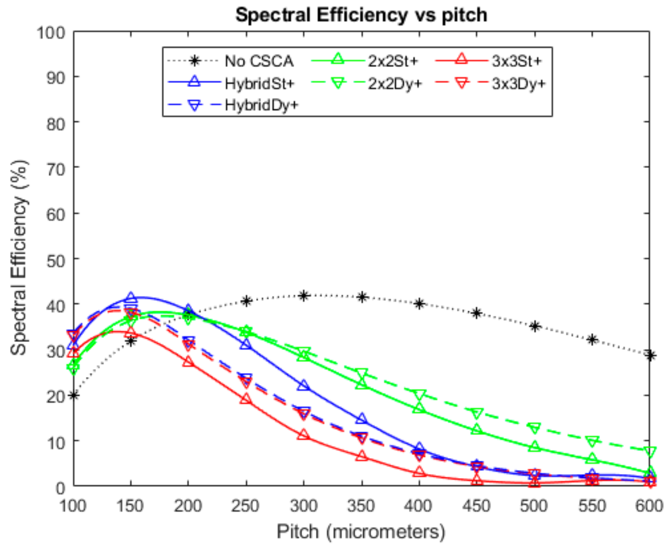
</td><td>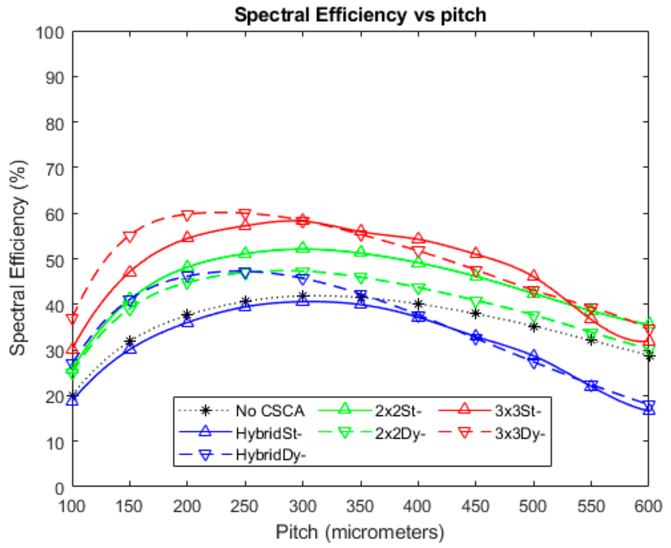
</td></tr>
<tr><td>10^9 photons mm^{-2} s^{-1}</td><td>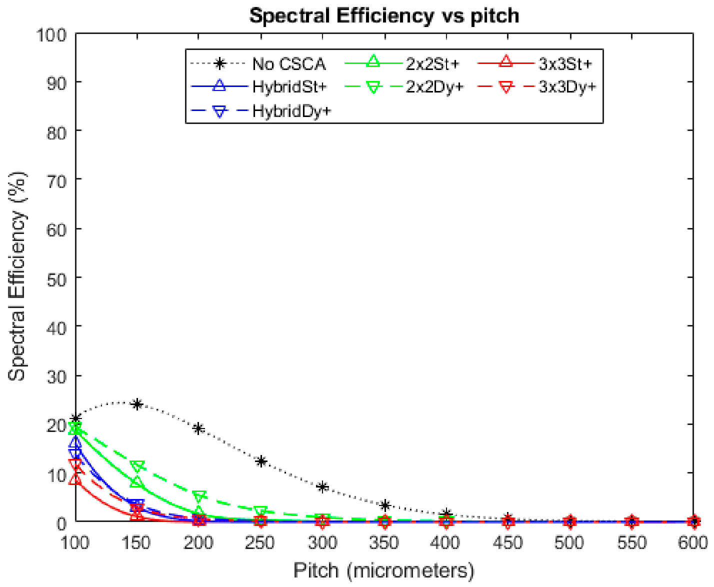
</td><td>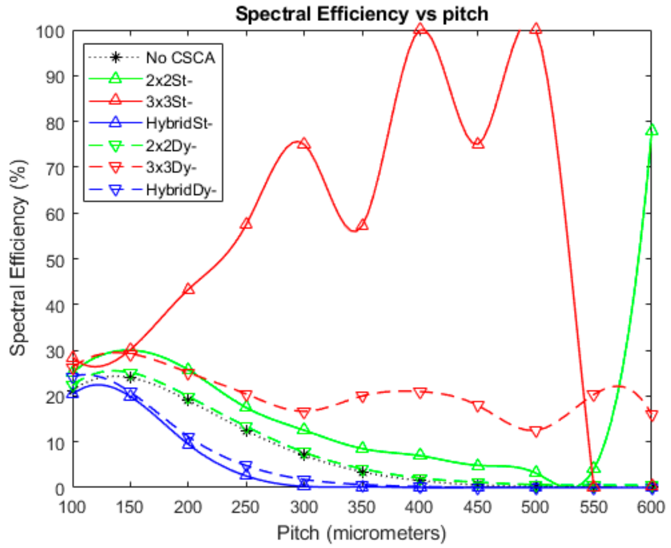
</td></tr>
</table>

<u>Total counts in photopeak bin</u>

Flux	Add	Sub

10^6 photons mm^{-2} s^{-1}	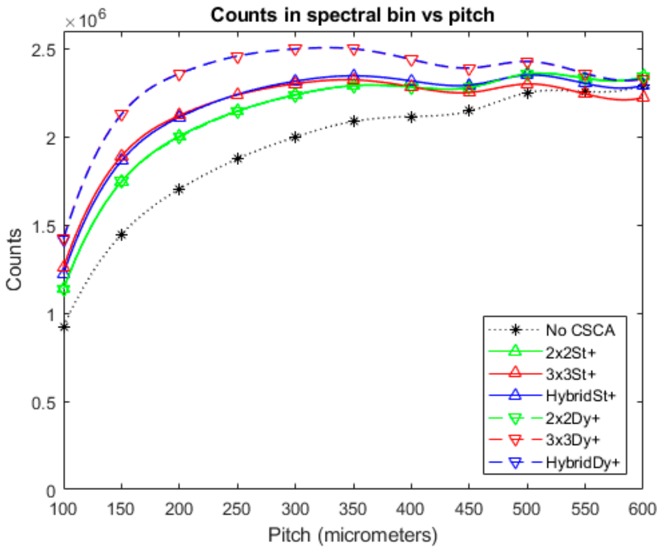	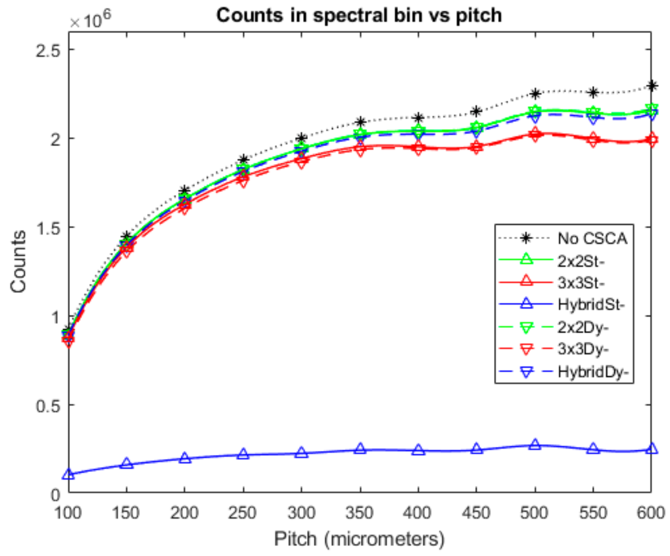
10^7 photons mm^{-2} s^{-1}	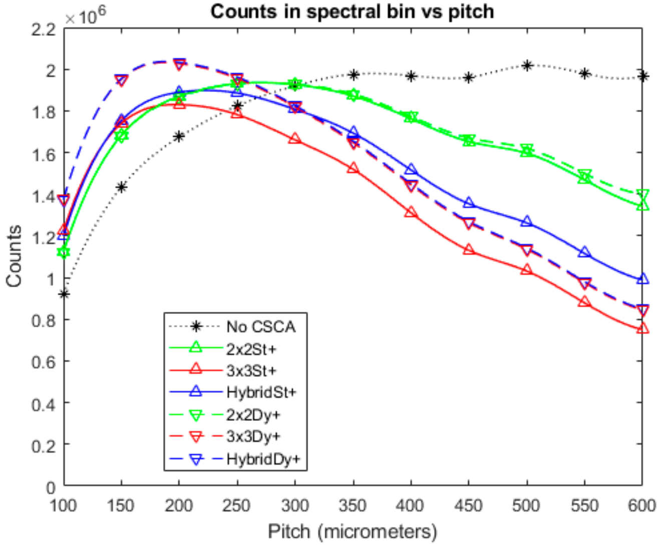	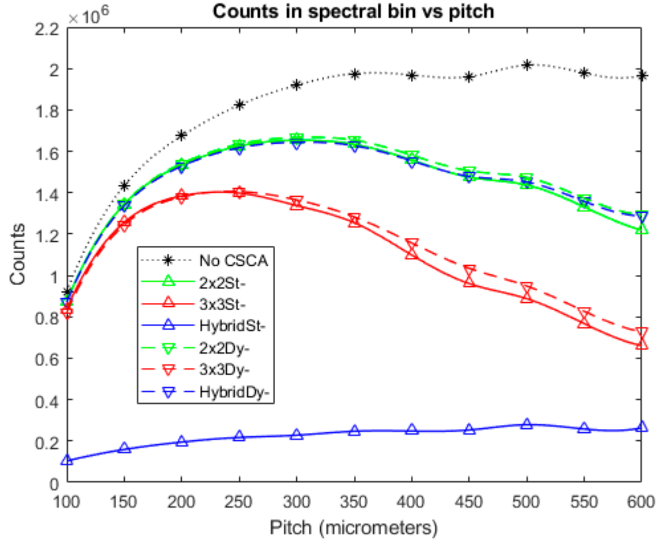

<table>
<tr>
<td>10^8 photons mm^{-2} s^{-1}</td>
<td>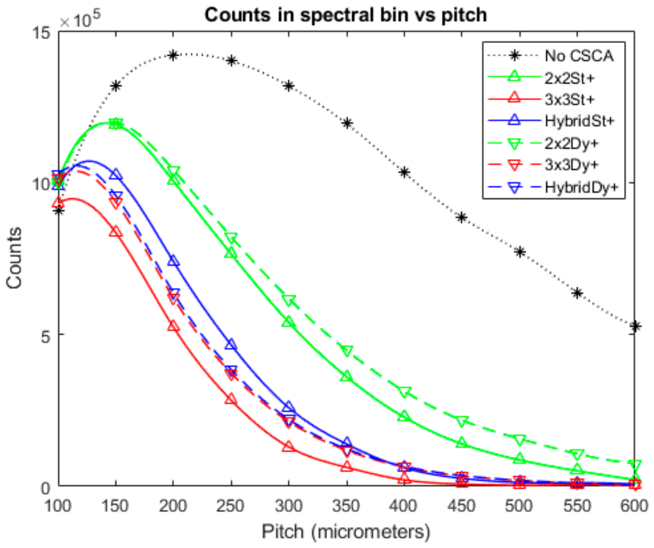
</td>
<td>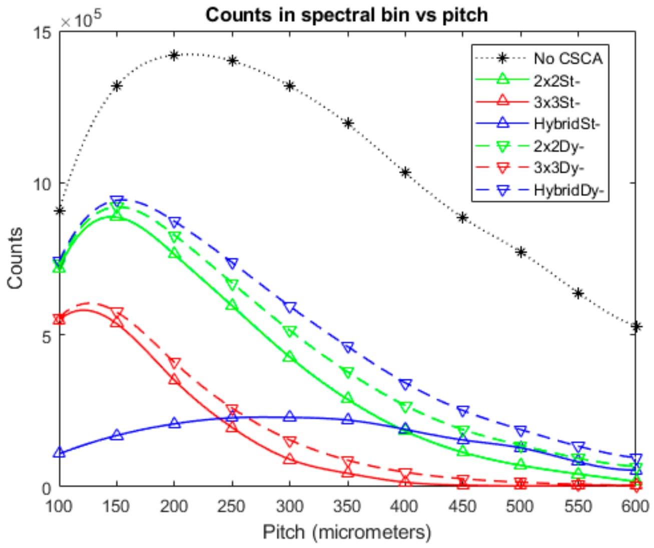
</td>
</tr>
<tr>
<td>10^9 photons mm^{-2} s^{-1}</td>
<td>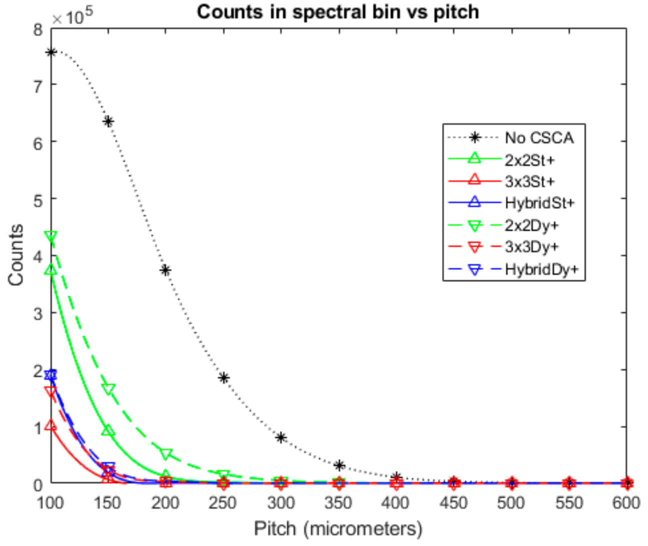
</td>
<td>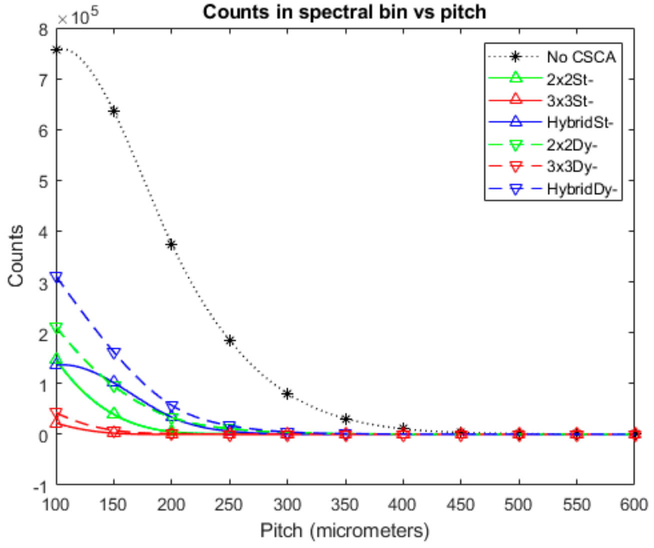
</td>
</tr>
</table>

Figure S1: Plots showing how APE, ADE, BSE, RCC, ‘total counts in coincidence bin’ and ‘total counts in photopeak bin’ vary as a function of pixel pitch for all x-ray fluxes simulated in this work.
